# Combinatorial Synthesis, Screening, and Binding Studies of Highly Functionalized Polyamino-amido Oligomers for Binding to Folded RNA

**DOI:** 10.1155/2012/971581

**Published:** 2012-08-21

**Authors:** Jonathan K. Pokorski, Daniel H. Appella

**Affiliations:** Laboratory of Bioorganic Chemistry, NIH, NIDDK, Bethesda, MD 20892, USA

## Abstract

Folded RNA molecules have recently emerged as critical regulatory elements in biological pathways, serving not just as carriers of genetic information but also as key components in enzymatic assemblies. In particular, the transactivation response element (TAR) of the HIV genome regulates transcriptional elongation by interacting specifically with the Tat protein, initiating the recruitment of the elongation complex. Preventing this interaction from occurring *in vivo* halts HIV replication, thus making RNA-binding molecules an intriguing pharmaceutical target. Using **α**-amino acids as starting materials, we have designed and synthesized a new class of polyamino-amido oligomers, called PAAs, specifically for binding to folded RNA structures. The PAA monomers were readily incorporated into a 125-member combinatorial library of PAA trimers. In order to rapidly assess RNA binding, a quantum dot-based fluorescent screen was developed to visualize RNA binding on-resin. The binding affinities of hits were quantified using a terbium footprinting assay, allowing us to identify a ligand (SFF) with low micromolar affinity (*k*_*d*_ = 14 *μ*M) for TAR RNA. The work presented herein represents the development of a flexible scaffold that can be easily synthesized, screened, and subsequently modified to provide ligands specific for binding to folded RNAs.

## 1. Introduction

Elucidation of the role played by small RNA molecules in the context of viral life cycles has led to the identification of new drug targets that fall outside the realm of traditional drug design [1]. In particular, the transactivation response element (TAR) of HIV is critical in regulating transcriptional elongation of the HIV genome [2]. TAR is a highly conserved stem-loop RNA located at the 5′-end of viral transcripts. For elongation to occur, the HIV transactivator protein (Tat) must first bind to TAR [2, 3]. The ensuing Tat-TAR complex is then responsible for recruitment of the positive transcription elongation factor complex, pTEFb, resulting in processive elongation [4]. The Tat-TAR complex is a particularly attractive drug target because it is highly conserved and difficult for the virus to develop resistance, which is a problem that hinders the effectiveness of conventional HIV therapies [5].

The most effective strategies to inhibit Tat-TAR formation rely upon mimicking the binding region of the Tat protein. Initial attempts to design TAR-binding molecules focused on dissecting the Tat peptide and synthesizing peptides corresponding to the highly basic, arginine rich region of Tat that binds to the trinucleotide bulge of TAR [6]. The truncated Tat peptides are able to compete with Tat *in vitro* for binding to TAR, however peptides are typically not viable drug candidates due to low bioavailability and stability [7]. Further studies have sought to mimic the Tat peptide with nonnatural oligomers. For instance, a nonamer peptoid was developed that had significant potency against the Tat-TAR interaction [8, 9]. Alternatively, cyclic peptides, and peptides comprised of D-amino acids had similar effects [10–12]. All of these molecules share a common feature in that they display highly cationic sidechains. We felt that the high degree of cationic charge associated with these molecules would lead to promiscuous RNA binders. Thus, we sought to create molecules that could retain similar binding properties while minimizing cationic charge about the periphery of the scaffold.

**Figure 1:**
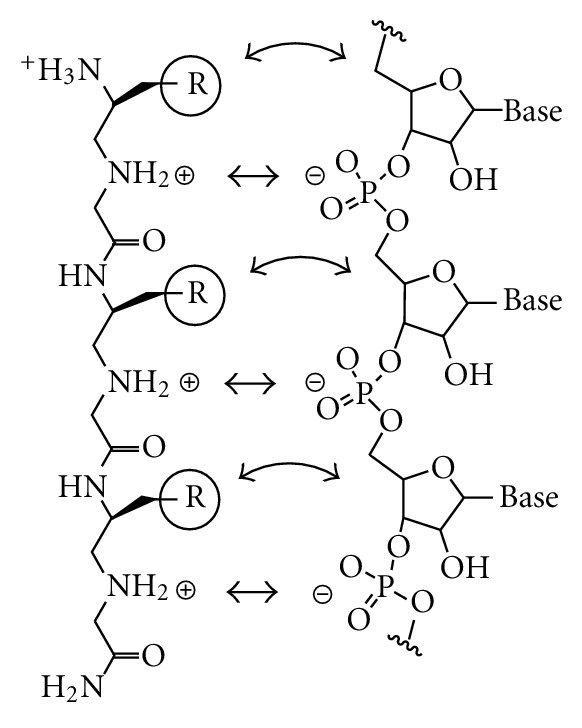
Structural comparison between PAA and RNA showing potential salt-bridges between ammonium groups in the PAA and the phosphodiester backbone of the RNA (straight double-headed arrows) and potential hydrophobic or hydrogen-bonding interactions between the sidechains of the PAA (R) and the RNA (curved double-headed arrow).

Initial results from our lab showed that highly functionalized polyamines were able to serve as TAR-binding molecules specific for the trinucleotide bulge [13]. Our design strategy served to relegate cationic charge to the interior of the polyamines to decrease nonspecific charge-charge interactions, while projecting sidechains out from the backbone to direct specificity. The first generation of polyamines showed promising results, yielding a polyamine that bound TAR specifically and with a *K*_*D*_ of ~6 *μ*M at the bulge. These polyamines, however, suffered from an inefficient oligomer synthesis that was not possible to develop into a combinatorial library. Due to the limited structural information available on TAR-binding molecules, we felt that expanding our strategy to allow for combinatorial library synthesis and subsequent screening was more likely to yield positive results. As such, we designed a new class of molecules, polyamino-amido oligomers (PAAs), that could be synthesized through modified solid phase peptide synthesis. The design principle of these molecules is illustrated in Figure 1. The secondary amines in the backbone, which should be protonated at physiological pH, are spaced at intervals corresponding to the spacing of the phosphate groups of the RNA backbone in order to form ammonium-phosphate salt bridges. From this general backbone scheme, amino acid derived sidechains can be introduced to direct binding specificity. While molecular modeling is often used as a tool for drug discovery, we felt that diversity-oriented approaches were more likely to yield positive results due to the dynamic nature of TAR RNA and the limited rational-based information available for design of small-molecule RNA binders. Furthermore, we wanted to reduce the number of highly exposed cationic charges that are prevalent in most RNA-binding small molecules which convey high binding affinity but also afford low specificity. In this paper, we describe the synthesis of the monomeric building blocks for PAAs as well as a parallel library synthesis. A quantum dot-based screen was implemented for an on-bead screening of the library for TAR binding. The binding affinity of hits was quantified using RNA footprinting assays. 

## 2. Materials and Methods

### 2.1. General Procedure A: Weinreb Amide Synthesis

An Fmoc-protected amino acid (10 mmol) and DIEA (1.6 mL) were dissolved in dry CH_2_Cl_2 _(50 mL) and cooled to 0°C. EDC (2.4 g, 12 mmol) and HOBt (1.8 g, 12 mmol) were added to the reaction. The reaction mixture was stirred at 0°C for 10 minutes to pre-activate the carboxylic acid. After 10 minutes, N,O-dimethylhydroxylamine hydrochloride (1.2 g, 12 mmol) and DIEA (2.0 mL) were added to the reaction mixture. The reaction was allowed to slowly warm to room temperature and was stirred for a total of 16 hours. The reaction was then transferred to a separatory funnel with CH_2_Cl_2_ (100 mL) and washed with 2 M HCl (3x, 50 mL), saturated aqueous NaHCO_3 _(2x, 50 mL), and brine (2x, 50 mL). The organic layer was dried over sodium sulfate and solvent was removed under reduced pressure to yield pure Weinreb amides as solid white foams.

### 2.2. General Procedure B: Reduction of Weinreb Amides to *α*-Amino Aldehydes

Dry THF (50 mL) and Weinreb amide (5 mmol) were added to an oven dried 250 mL round bottom flask, placed under N_2_, and cooled to 0°C in an ice bath. Lithium aluminum hydride (250 mg, 6.25 mmol) was slowly added to the solution over a period of approximately 30 seconds. The reaction was stirred vigorously at 0°C for 60 minutes and then slowly quenched with a 1 M solution of NaHSO_4_ (50 mL). The biphasic mixture continued to stir at 0°C for 10 minutes at which point it was transferred to a separatory funnel using EtOAc (50 mL) and brine (50 mL). The aqueous layer was extracted with EtOAc (75 mL) and the combined organic layers were washed with 1.5 M HCl (2x, 50 mL), saturated aqueous NaHCO_3_ (2x, 50 mL), brine (2x, 50 mL). The organic layer was dried over sodium sulfate and concentrated under reduced pressure. The resulting amino aldehydes were taken forward without additional purification.

### 2.3. General Procedure C: Reductive Amination to Form the PAA Backbone

An Fmoc-protected *α*-amino aldehyde (4.5 mmol) was dissolved in dry CH_2_Cl_2_ (30 mL) at room temperature. To the stirring solution was added a benzyl protected amino acid hydrochloride (4.9 mmol) and DIEA (0.85 mL). NaBH(OAc)_3_ (6.3 mmol) was immediately added to the reaction mixture and stirred vigorously for 75 minutes. The reaction was quenched with a mixture of saturated aqueous K_2_CO_3_ (10 mL) and saturated aqueous NaHCO_3_ (30 mL) and allowed to stir for an additional 10 minutes. The biphasic mixture was transferred to a separatory funnel and extracted with CH_2_Cl_2_ (3x, 30 mL). The organic layers were combined and dried over sodium sulfate, filtered, and concentrated under reduced pressure. The crude product was purified using a biotage flash chromatography system (40+M column, 60 : 40 Hexanes: EtOAc).

### 2.4. General Procedure D: Boc Protection of Secondary Amine in the PAA Backbone

The PAA secondary amine backbone (2.0 mmol) was dissolved in dry CH_2_Cl_2_ (40 mL) and stirred at room temperature. To the solution was added di-tert-butyl dicarbonate (0.9 g, 4 mmol) and DIEA (0.6 mL, 2 mmol). The reaction was allowed to stir for 48 hours and was then transferred to a separatory funnel with CH_2_Cl_2 _(50 mL). The reaction mixture was washed with 1 M HCl (2x, 50 mL), saturated aqueous NaHCO_3_ (2x, 50 mL), and brine (2x, 50 mL). The organic layer was dried over sodium sulfate and concentrated under reduced pressure. The crude product was purified using a biotage flash chromatography system (40+M column, 80 : 20 Hexanes: EtOAc).

### 2.5. General Procedure E: Hydrogenolysis of Benzyl Ester to Yield PAA Monomers

A Parr flask was purged with N_2_ and then charged with 10% palladium on carbon. The palladium catalyst was wetted with a minimum amount of methanol (~5 mL) while under an N_2_ atmosphere. The PAA-monomer ester (1.5 mmol) was dissolved in a minimal amount of methanol (typically ~20 mL) and added to the Parr flask. The flask was placed under an H_2_ atmosphere (40 psi) and shaken on a Parr shaker for 2 hours. The reaction mixture was then filtered through a bed of celite to remove the palladium from the mixture. The resulting solution was concentrated under reduced pressure to yield an off-white solid as the crude product. The crude product was purified using a biotage flash chromatography system (40+M column, 0%–5% MeOH gradient in CH_2_Cl_2_).

### 2.6. Screen for TAR RNA-Binding to PAA Library Members

Several beads from each well of the PAA library were transferred to a 384-well filter plate, keeping their spatial separation and orientation intact. The beads were first washed with water (5x, 50 *μ*L), then 1x TK buffer (50 mM Tris, 20 mM KCl, 0.1% Triton X-100, pH 7.4; 4x–50 *μ*L). To each well, BSA (0.1 mg/mL) was added in 1x TK buffer (20 *μ*L) and agitated with mechanical shaking for 60 minutes at room temperature. The microplate was drained under vacuum and washed with 1x TK buffer (3x, 50 *μ*L). Following BSA blocking, bulge mutant TAR in 1x TK buffer (2.5 *μ*M, 20 *μ*L/well) was added to each individual well. The library was incubated with bulge mutant TAR for 24 hours at 4°C before being drained under vacuum. Immediately following solvent removal, a mixture of bulge mutant TAR (2.5 *μ*M) and 5′-biotin labeled TAR (Dharmacon, 250 nM) in 1x TK buffer (20 *μ*L/well) were introduced to the library. (Note: RNA was snap-cooled by heating at 95°C for 5 minutes followed by an immediate transfer to dry ice for 5 minutes to promote hairpin formation.) The library was incubated with this solution for 2.5 days at 4°C, drained, and washed with water. To each well a solution of Qdot605 (50 nM, 15 *μ*L/well) in 1x TK buffer was added and agitated at room temperature for 3 hours. The solution was drained and each well was washed with 1x TK (3x–50 *μ*L), followed by a 2 hour wash with 1x TK buffer and drainage under vacuum. The library was then visualized using a fluorescent microscope equipped with a triple bandpass filter. Beads that appeared red or orange under the microscope were selected for further characterization while those that were green were disregarded.

## 3. Results and Discussion

### 3.1. Synthesis of Polyamines

**Scheme 1:**
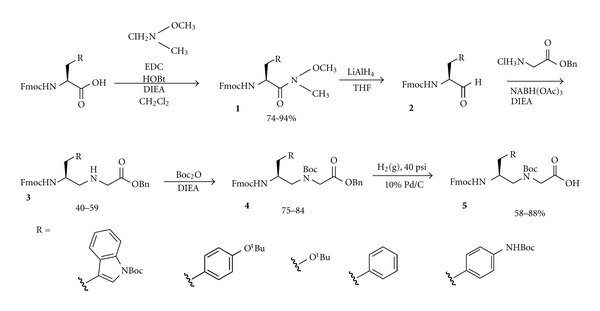
Synthesis of PAA monomers.

Polyamine monomers were synthesized starting from commercially available Fmoc-protected amino acids utilizing a solution-phase reductive amination strategy. Initially we sought to synthesize five polyamine monomers starting from orthogonally protected serine, tryptophan, tyrosine, 4-amino-phenylalanine, and phenylalanine (Scheme 1). The monomers were selected based on their likelihood to interact with folded RNA, thus amino acids capable of *π*-stacking, hydrophobic interactions, and hydrogen bonding were selected. We chose to exclude certain high-affinity moieties, such as guanidinium groups, due to their strongly cationic nature and tendency to interact non-specifically. This choice aligns with our design strategy, in which positive charge is sequestered to the backbone to reduce non-specific charge-charge interactions with the RNA backbone. The Fmoc-protected amino acid was first converted to the Weinreb amide ***(1)*** in high yield under EDC-mediated amide bond forming conditions. Subsequently, ***1*** was reduced to the corresponding aldehyde ***(2)*** using lithium aluminum hydride. The aldehyde product was taken forward without additional purification to the reductive amination step where ***2*** was condensed with the hydrochloride salt of benzyl glycinate. After imine formation, reduction with sodium triacetoxy borohydride provided ***3***. Following chromatographic purification, the secondary amine was protected with a Boc group to afford the PAA monomer ester. Hydrogenolysis of the benzyl ester yielded the PAA monomer ***(5)***.

From these five monomers, a library of 125 PAA trimers was synthesized in parallel on solid support. The synthesis was designed to be compatible with the development of an on-bead screen for RNA binding. In order to create a library suitable for aqueous screening conditions, Tentagel-NH_2_ resin was chosen as a synthetic platform due to its unique ability to swell in both aqueous and organic solvents. In addition, the library synthesis was performed in 96-well filter plates to provide a physical separation between distinct PAAs. The strategy outlined provided an accessible synthetic platform that negated the need for molecular deconvolution during the screening process. The synthesis began by functionalizing the resin with an Fmoc-*β*-alanine spacer. The spacer was deprotected and PAA trimers were synthesized through HATU-mediated solid phase peptide synthesis (see supplementary material available online at doi:10.1155/2012/971581). Upon completion of the trimers, a global deprotection of the backbone and sidechain protecting groups afforded a 125-member library of resin-bound PAA trimers.

### 3.2. On Bead Screen for TAR Binding

**Figure 2:**
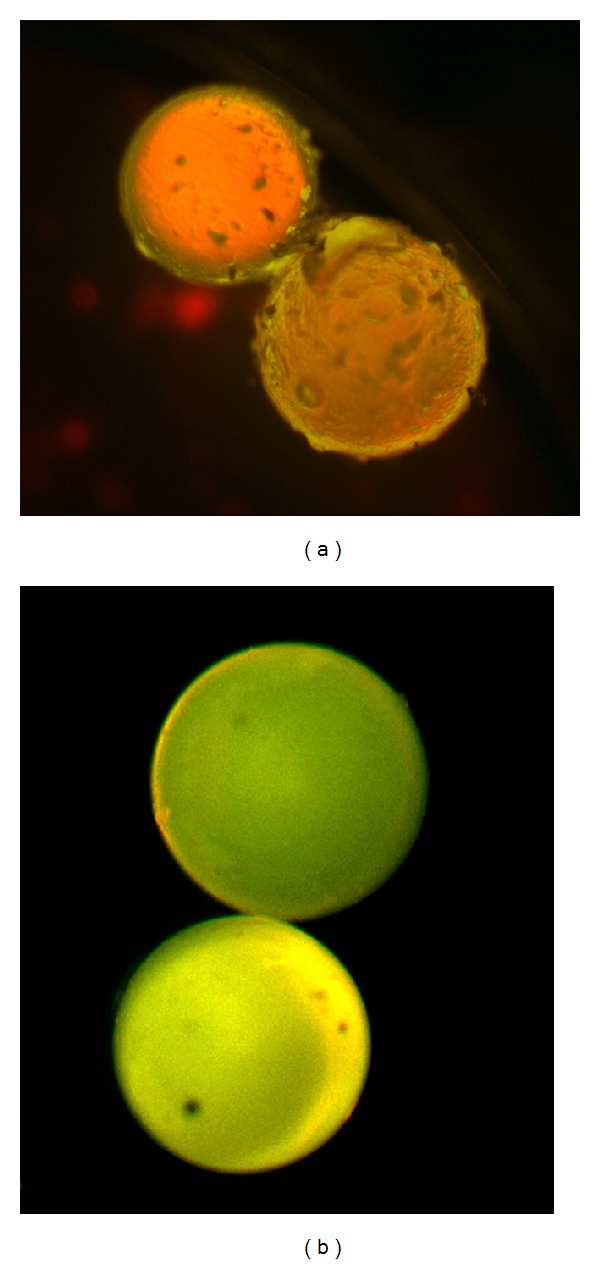
Representative pictures from screen of PAA library. The left hand picture represents a positive hit, while the picture on the right is a negative result within our screen.

Initially we aimed to develop a simple fluorescent screening procedure for assessing TAR binding to our resin-bound PAAs. To this end, we adapted protocols developed by the Rana and Kodadek laboratories to our system [12, 14–16]. Since on-bead binding and high activity in solution are not necessarily correlated, our assay design was carefully chosen based on validated literature results. The incubation conditions mimicked those reported by Rana [12], whose bead-based assays showed strong positive correlation to appropriate off-bead activity measurements. The PAA-containing beads were first incubated with a solution of BSA to block any nonspecific binding to the bead surface. Subsequently, the library was incubated with a bulge mutant TAR construct containing a single base bulge, rather than the wildtype trinucleotide bulge. The bulge mutant acted as a competitive inhibitor to exclude compounds that were not specific for the bulge region of TAR. Next, 5′-biotinylated wildtype TAR was added to the resin in the presence of an excess of bulge mutant TAR. Binding events were visualized using a fluorescent microscope equipped with a triple bandpass filter after the addition of streptavidin-coated quantum dots (Qdot605). This method allowed for the visualization of positive beads as red in color where beads containing nonbinding polyamines were seen as green under the microscope (Figure 2). From this initial screen the six brightest beads were selected, as determined by visual inspection and verified independently by a second researcher, and resynthesized on a larger scale to allow determination of binding constants. To describe the PAAs, the standard one-letter amino acid abbreviations are used to describe the sequence of sidechains with the understanding that in this paper the backbone is represented by the chemical structure in Figure 1 for a PAA backbone. The six PAAs selected based on the screen were SFF, YFF, FFF, SYS, YSF, and FYY. 

### 3.3. Terbium Footprinting Experiments

**Figure 3:**
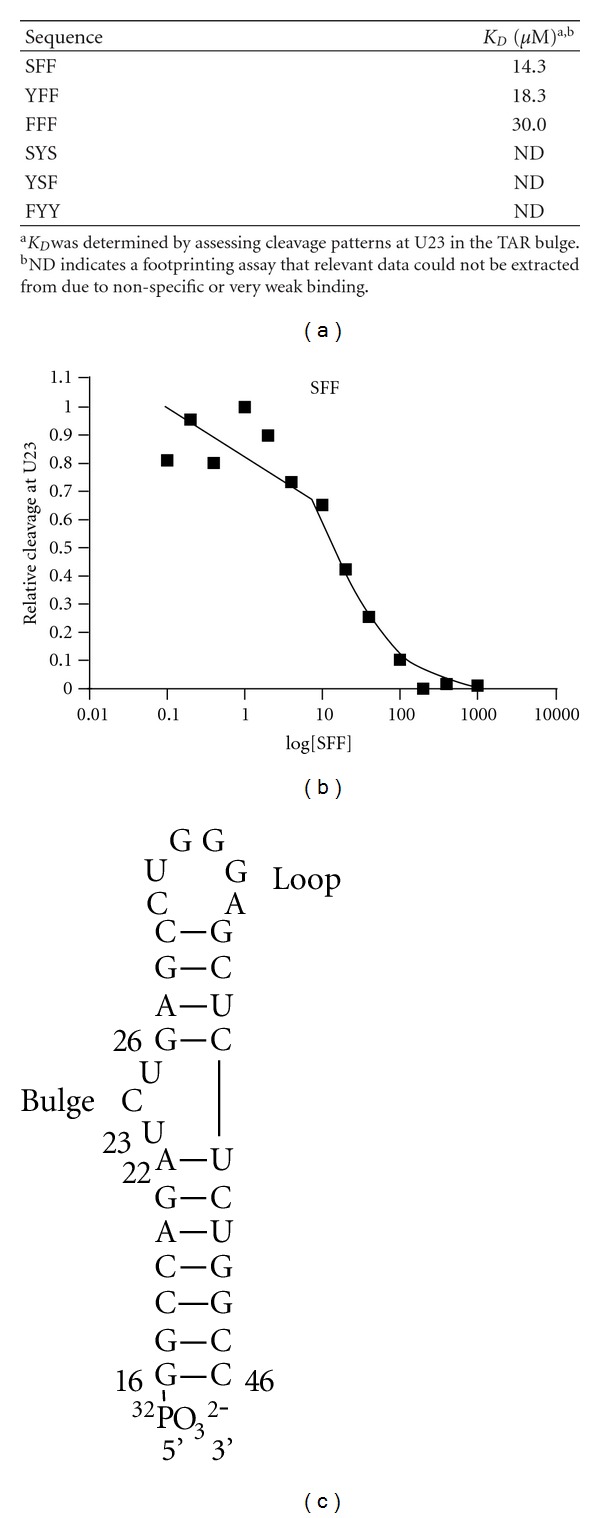
(a) Sequences of PAA “hits” from the initial screen and their corresponding dissociation constants for the bulge region of TAR. (b) Binding curve for SFF as determined by quantifying changes in cleavage at U23 as a function of SFF concentration (c) Structure of TAR RNA hairpin.

**Figure 4:**
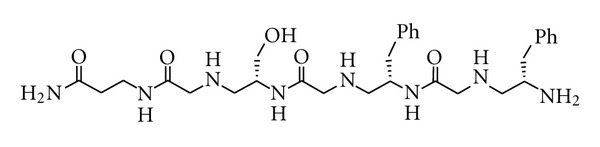
Structure of SFF ligand.

Previous work in our lab found RNA footprinting studies using terbium (III) ions as an RNA cleavage agent to be a reliable method for quantification of polyamine binding to TAR RNA [13, 17]. The six selected PAAs were synthesized, purified by reversed phase HPLC, and quantified by UV absorbance. The PAAs were then titrated into buffered solutions containing TAR up to 1 mM concentrations and effects on RNA cleavage patterns were assessed as a function of PAA concentration via denaturing gel electrophoresis. The results of these experiments identified the sequence XFF as a binding motif specific for the bulge region of TAR. Three of the six ligands selected showed appreciable binding affinity for the bulge region, with SFF, YFF, and FFF exhibiting binding constants in the low micromolar range (Figure 3). The best ligand derived from our initial screen was SFF (Figure 4), exhibiting a *K*_*D*_ of 14 *μ*M for the bulge region. Additionally, no binding was observed for the loop region of the TAR RNA, suggesting that our screen effectively selected for molecules that specifically interacted with the desired bulge region rather than nonspecific RNA binders (see supplementary material). 

### 3.4. Alanine Scan

We next sought to probe the importance of sidechain interactions in an effort to define a minimal binding motif for the bulge region. The most direct way for us to probe this question was to substitute each sidechain in our best ligand (SFF) with a moiety deemed to be unlikely to interact with the RNA. In analogy to alanine scans in peptides, we synthesized the alanine-derived PAA monomer and iteratively replaced each monomer in the SFF PAA with one bearing a methyl sidechain [18]. The three PAAs were synthesized and subjected to terbium footprinting assays. We found that all three sidechain replacements yielded compounds with very low affinity for the TAR target, thus leading us to conclude that all three sidechains were critical to maintain binding affinity. 

### 3.5. Secondary Library Synthesis and Screen

**Figure 5:**
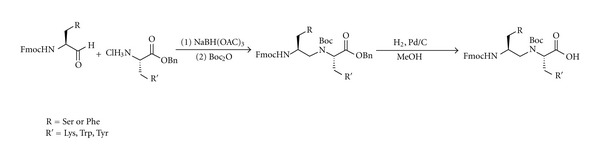
Synthesis of secondary library monomers.

The PAA scaffold was intentionally designed such that modifications to the backbone could be easily installed in order to determine structural activity relationships (SARs). Therefore, modification to the backbone of the SFF core motif could lead to enhanced binding affinity for TAR. In this vein, we aimed to synthesize a library of compounds based on the SFF core but bearing sidechains in place of the glycine subunit of the PAA monomers. Six new monomers were synthesized, where the key step involved condensation between serine or phenylalanine aldehydes and the hydrochloride salts of either tryptophan, tyrosine, or lysine (Figure 5). The new monomers and the original serine and phenylalanine monomers were incorporated into a 64-member PAA library, where all library members contained the previously identified SFF core. The library was again subjected to the Qdot based screen and the six brightest hits were chosen for further characterization. Unfortunately, all PAA derived from the second screen showed either very weak affinity for TAR or no affinity at all in the footprinting assays (see supplementary material). 

As biological knowledge has advanced, RNA has emerged as a viable pharmaceutical target. To date, there are few *de novo* designed molecules that are capable of specifically interacting with folded RNA. In this paper, we have presented a class of readily synthesized oligomers that can specifically bind to the bulge region of folded TAR RNA. In developing these molecules, we implemented a quantum dot based fluorescent screening protocol that readily identified ligands for RNA. Our screening protocol allowed for the determination of a minimal binding motif from the initial PAA library. Quantification of binding affinity via footprinting analysis yielded a low micromolar binder of TAR RNA specific for the bulge region. We anticipate that further understanding of RNA-mediated biological pathways will serve to validate RNA as a viable drug target. Consequently, the ability to rapidly develop drug candidates that are able to distinguish between subtle differences in RNA secondary structure will be of the utmost importance. We feel that the methods and results presented in this work represent a step toward developing a general class of RNA binding molecules that can suit this purpose (see supplementary material).

## Supplementary Material

Detailed protocols and data for the solid phase synthesis of PAA trimers, terbium footprinting assays, and monomer characterization are provided.
