# Supplementary material for: Combinatorial Synthesis, Screening, and Binding Studies of Highly Functionalized Polyamino-amido Oligomers for Binding to Folded RNA
Source: J Nucleic Acids. 2012 Aug 21;2012:971581. doi: 10.1155/2012/971581 (PMC3432390; doi:10.1155/2012/971581)
Supplement: Supplementary file 1 — Detailed protocols and data for the solid phase synthesis of PAA trimers, terbium footprinting assays, and monomer characterization are provided. [file 971581.f1.pdf]

Supporting Information for:

Synthesis, Screening and Binding Studies of Highly Functionalized Polyamino-amido Oligomers for Binding to Folded RNA

Jonathan K. Pokorski and Daniel H. Appella

General Methods: Proton nuclear magnetic resonances (**^{1}H NMR**) were recorded in deuterated solvents on a Mercury 300 (300 MHz) spectrometer. Chemical shifts are reported in parts per million (ppm, δ) relative to tetramethylsilane (δ 0.00) or residual protio solvent (DMSO, δ 2.50). ^{1}H NMR splitting patterns are designated as singlet (s), doublet (d), triplet (t), or doublet of doublets (dd). Splitting patterns that could not be easily interpreted or visualized were recorded as multiplet (m) or broad (br). Coupling constants are reported in Hertz (Hz). Proton-decoupled carbon (**^{13}C-NMR**) spectra were recorded on a Mercury 300 (75 MHz) spectrometer and are reported in ppm using the solvent as an internal standard (DMSO, δ 39.52). The accurate mass electrospray ionization mass spectra (**ESI-MS**) were obtained on a Waters LCT Premier time-of-flight (TOF) mass spectrometer. The instrument was operated in W-mode at a nominal resolution of 10000. The electrospray capillary voltage was 2KV and the samplecone voltage was 60 volts. The desolvation temperature was 275 °C and the desolvation gas was nitrogen with a flow rate of 300 L/hr. Accurate masses were obtained using the internal reference standard method. The sample was introduced into the mass spectrometer via the direct loop injection method. Both positive and negative ion accurate mass data were achieved simply by reversing the instrument's operating polarity. Methylene chloride (CH_2Cl_2) was purified by passing solvent through a column of activated alumina on a solvent purification system built by Glass Contour prior to use.[1] Dimethylformamide (DMF) was first passed through activated alumina and was additionally purified with an isocyanate scavenger column prior to use. All other solvents were purchased from Sigma-Aldrich and used without further purification. All reactions were carried out under positive nitrogen pressure unless otherwise noted. Flash column chromatography was performed using a Biotage Flash 12+ apparatus using Biotage Si 40+M columns. Solvent mixtures for column chromatography are reported in volume/volume ratios. Rink resin was purchased from Advanced ChemTech. All other reagents were purchased from Sigma-Aldrich. PAA oligomers were purified by reversed-phase HPLC using an Agilent 1100 series preparative HPLC with UV detection at 220 nm equipped with a Vydac C18 semi-prep column (10 mm x 250 mm). Solution A was 0.05% TFA in water and solution B was 0.05% TFA in acetonitrile. A typical elution gradient went from 100% A to 65% A over 45 minutes with a flow rate of 5 mL/min.

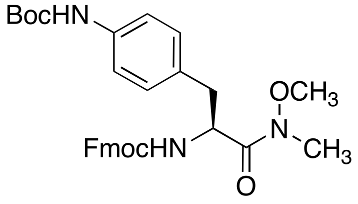

Fmoc-4-aminophenylalanine(Boc) Weinreb Amide: ^{1}H NMR ($CDCl_3$, 300 MHz) δ 1.51 (s, 9H), 3.01 (m, 2H), 3.18 (s, 3H), 3.68 (s, 3H), 4.21 (t, *J* = 7.5 Hz, 1H), 4.34 (m, 2H), 5.0 (br, 1H), 5.48 (d, *J* = 9 Hz, 1H), 6.44 (s, 1H), 7.08 (d, *J* = 8.1 Hz, 2H), 7.30 (m, 4H), 7.39 (t, *J* = 7.5 Hz, 2H), 7.56 (t, *J* = 7.2 Hz, 2H), 7.75 (d, *J* = 7.2 Hz, 2H). 94 % Yield.

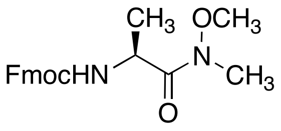

Fmoc-Ala Weinreb Amide: ^{1}H NMR ($CDCl_3$, 300 MHz) δ 1.39 (d, *J* = 6.9 Hz, 3H), 3.24 (s, 3H), 3.79 (s, 3H), 4.23 (t, *J* = 6.6 Hz, 1H), 4.37 (d, *J* = 7.2 Hz, 2H), 4.77 (t, *J* = 7.8 Hz, 2H), 5.60 (d, *J* = 8.7 Hz, 1H), 7.32 (t, *J* = 7.5 Hz, 2H), 7.41 (t, *J* = 7.5 Hz, 2H), 7.62 (dd, *J* = 2.7, 6.9 Hz, 2H), 7.77 (d, *J* = 7.2 Hz, 2H). 74 % Yield.

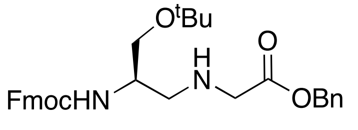

Gly-Ser(^{t}Bu) Backbone: ^{1}H NMR ($CDCl_3$, 300 MHz) δ 1.18 (s, 9H), 1.83 (br, 1H), 2.83 (m, 2H), 3.48 (m, 4H), 3.81 (br, 1H), 4.24 (t, *J* = 6.6 Hz, 1H), 4.37 (br, 2H), 5.18 (s, 2H), 5.42 (d, *J* = 4.5 Hz, 1H), 7.36 (m, 9H), 7.61 (d, *J* = 7.2 Hz, 2H), 7.77 (d, *J* = 7.5 Hz, 2H). 68 % Yield.

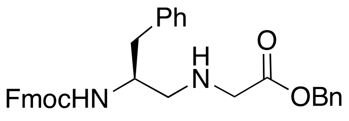

Gly-Phe Backbone: ^{1}H NMR ($CDCl_3$, 300 MHz) δ 1.65 (s, 1H), 2.65 (m, 2H), 2.84 (m, 2H), 3.43 (q, *J* = 29.1, 10.5 Hz, 2H), 3.95 (br, 1H), 4.20 (t, *J* = 6.9 Hz, 1H), 4.41 (m, 2H), 5.10 (br d, 1H), 5.16 (s, 2H), 7.31 (m, 12H), 7.39 (t, *J* = 7.5 Hz, 2H), 7.57 (t, *J* = 6.9 Hz, 2H), 7.76 (d, *J* = 7.2 Hz, 2H). 63 % Yield.

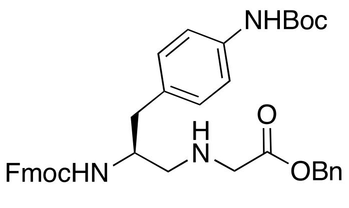

Gly-(4-NH_2)Phe Backbone: ^{1}H NMR ($CDCl_3$, 300 MHz) δ 1.51 (s, 9H), 1.60 (s, 1H), 2.62 (br, 2H), 2.79 (m, 2H), 3.41 (q, *J* = 10.2 Hz, 2H), 3.90 (br, 2H), 4.19 (t, *J* = 6.3 Hz, 1H), 4.38 (m, 2H), 5.08 (br d, 1H), 5.15 (s, 2H), 6.43 (s, 1H), 7.08 (d, *J* = 6 Hz, 2H), 7.27 (m, 2H), 7.34 (m, 9H), 7.57 (t, *J* = 6.6 Hz, 2H), 7.75 (d, *J* = 7.2 Hz, 2H). 83% Yield.

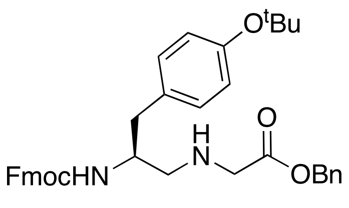

Gly-Tyr(^{t}Bu) Backbone: ^{1}H NMR ($CDCl_3$, 300 MHz) δ 1.30 (s, 9H), 2.63 (br, 2H), 2.79 (m, 2H), 3.41 (q, *J* = 11.5 Hz, 2H), 3.92 (br, 1H), 4.19 (t, *J* = 6.8 Hz, 1H), 4.38 (m, 2H), 5.09 (br d, 1H), 5.14 (s, 2H), 6.88 (d, *J* = 7.8 Hz, 2H), 7.04 (d, *J* = 7.5 Hz, 2H), 7.34 (m, 9H), 7.57 (d, *J* = 6.9 Hz, 2H), 7.75 (d, *J* = 7.8 Hz, 2H). 61 % Yield.

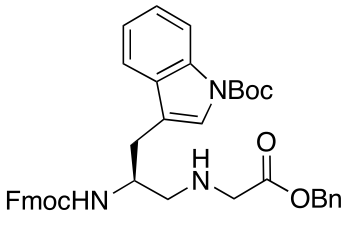

Gly-Trp(Boc) Backbone: 1H NMR (DMSO, 300 MHz) δ 1.54 (s, 9H), 2.65 (d, *J* = 5.7 Hz, 2H), 2.83 (m, 4H), 3.82 (br m, 1H), 4.18 (m, 3H), 5.12 (s, 2H), 7.32 (m, 11H), 7.49 (s, 1H), 7.59 (d, *J* = 7.5 Hz, 2H), 7.68 (d, *J* = 8.4 Hz, 1H), 7.87 (d, *J* = 7.8 Hz, 2H), 8.02 (d, *J* = 9 Hz, 1H). 52 % Yield.

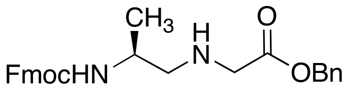

Gly-Ala Backbone: ^{1}H NMR ($CDCl_3$, 300 MHz) δ 1.27 (d, *J* = 5.4 Hz, 3H), 2.76 (br, 2H), 3.56 (q, *J* = 7.5 Hz, 2H), 3.85 (br, 1H), 4.34 (t, *J* = 6.6 Hz, 1H), 4.52 (br, 2H), 5.19 (br, 1H), 5.29 (s, 2H), 7.43 (m, 7H), 7.51 (t, *J* = 7.2 Hz, 2H), 7.72 (t, *J* = 6.3 Hz, 2H), 7.87 (d, *J* = 7.2 Hz, 2H). 33% Yield.

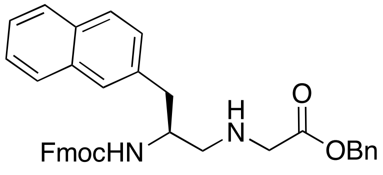

Gly-Nap Backbone: ^{1}H NMR ($CDCl_3$, 300 MHz) δ 2.71 (br, 2H), 3.02 (m, 2H), 3.45 (q, *J* = 10.5 Hz, 2H), 4.07 (br, 1H), 4.21 (t, *J* = 7.5 Hz, 1H), 4.43 (m, 2H), 5.16 (s, 2H), 5.21 (br, 1H), 7.29 (m, 2H), 7.35 (m, 8H), 7.47 (m, 2H), 7.57 (t, *J* = 8.7 Hz, 2H), 7.65 (s, 1H), 7.79 (m, 5H). 65% Yield.

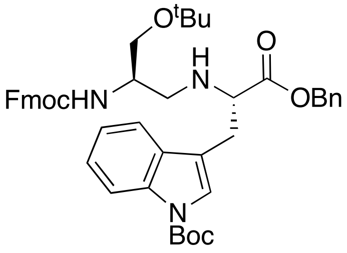

Trp(Boc)-Ser(^{t}Bu) Backbone: ^{1}H NMR (DMSO, 300 MHz) δ 1.01 (s, 9H), 1.59 (s, 9H), 2.28 (br, 1H), 2.69 (br, 1H), 2.97 (d, *J* = 6.9 Hz, 2H), 3.21 (d, *J* = 5.7 Hz, 2H), 3.50 (br m, 2H), 3.59 (br m, 2H), 4.23 (m, 3H), 5.00 (s, 2H), 7.04 (d, *J* = 8.1 Hz, 1H), 7.13 (dd, *J* = 3.9, 3.6 Hz, 2H), 7.28 (m, 6H), 7.43 (m, 3H), 7.57 (d, *J* = 7.8 Hz, 1H), 7.68 (d, *J* = 7.2 Hz, 2H), 7.88 (d, *J* = 7.8 Hz, 2H), 8.02 (d, *J* = 7.8 Hz, 1H). 64% Yield

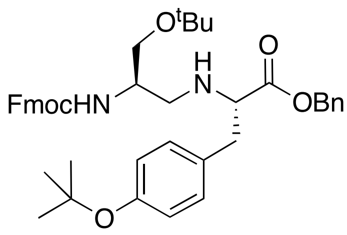

Tyr(^{t}Bu)-Ser(^{t}Bu) Backbone: ^{1}H NMR ($CDCl_3$, 300 MHz) δ 1.11 (s, 9H), 1.30 (s, 9H), 2.70 (br, 2H), 2.87 (m, 2H), 3.31 (m, 2H), 3.55 (br, 1H), 3.68 (br, 1H), 4.23 (t, *J* = 7.2 Hz, 1H), 4.35 (d, *J* = 6.9 Hz, 2H), 5.09 (d, *J* = 3.6 Hz, 2H) 5.33 (br, 1H), 6.86 (d, *J* = 8.4 Hz, 2H), 7.03 (d, *J* = 8.7 Hz, 2H), 7.27 (m, 7H), 7.39 (t, *J* = 7.2 Hz, 2H), 7.61 (d, *J* = 7.2 Hz, 2H), 7.76 (d, *J* = 7.5 Hz, 2H). 58 % Yield.

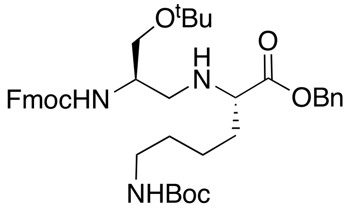

Lys(Boc)-Ser(^{t}Bu) Backbone: ^{1}H NMR ($CDCl_3$, 300 MHz) δ 1.16 (s, 9H), 1.35 (m, 3H), 1.43 (s, 9H), 1.63 (m, 3H), 2.70 (m, 2H), 3.03 (d, *J* = 6 Hz, 2H), 3.28 (br, 2H), 3.44 (m, 1H), 3.45 (m, 2H), 3.73 (br, 1H), 4.23 (t, *J* = 7.2 Hz, 1H), 4.37 (d, *J* = 7.5 Hz, 2H), 4.48 (br, 1H), 5.15 (d, *J* = 2.1 Hz, 2H), 5.31 (br, 1H), 7.34 (m, 7H), 7.39 (t, *J* = 7.2 Hz, 2H), 7.60 (d, *J* = 7.5 Hz, 2H), 7.76 (d, *J* = 7.5 Hz, 2H). 54% Yield.

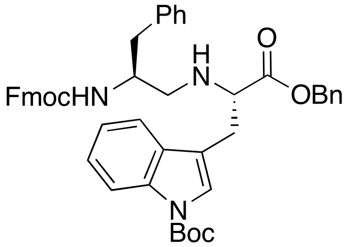

Trp(Boc)-Phe Backbone: ^{1}H NMR (CDCl$_3$, 300 MHz) δ 1.56 (s, 9H), 2.59 (m, 2H), 2.80 (m, 1H), 2.99 (t, *J* = 5.1 Hz 2H), 3.59 (br, 2H), 4.17 (m, 3H), 5.01 (s, 2H), 7.02 (d, *J* = 6.3 Hz, 1H), 7.14 (m, 4H), 7.27 (m, 10H), 7.40 (t, *J* = 7.2 Hz, 2H), 7.51 (s, 1H), 7.61 (t, *J* = 7.2 Hz, 2H), 7.88 (d, *J* = 7.8 Hz, 2H), 8.04 (d, *J* = 8.4 Hz, 1H). **ESI-MS:** expected mass 750.3543, observed mass 750.3557. 42% Yield.

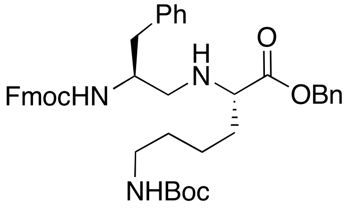

Lys(Boc)-Phe Backbone: ^{1}H NMR (CDCl$_3$, 300 MHz) δ 1.43 (s, 9H), 1.46 (m, 6H), 2.43 (br, 1H), 2.69 (m, 1H), 2.82 (br, 2H), 3.04 (m, 2H), 3.21 (br, 1H), 3.89 (br, 1H), 4.19 (t, *J* = 6.6 Hz, 1H), 4.31 (br, 1H), 4.45 (m, 2H), 5.00 (br, 1H), 5.12 (s, 2H), 7.30 (m, 14H), 7.55 (dd, *J* = 3, 4.2 Hz, 2H), 7.75 (d, *J* = 7.5 Hz, 2H). 55% Yield.

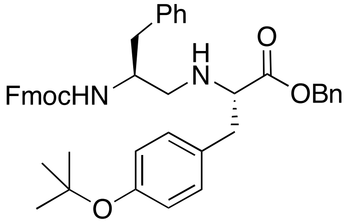

Tyr(^{t}Bu)-Phe Backbone: ^{1}H NMR (CDCl$_3$, 300 MHz) δ 1.31 (s, 9H), 2.48 (br, 2H), 2.77 (m, 2H), 2.98 (br, 2H), 3.86 (br, 1H), 4.21 (t, *J* = 7.2 Hz, 1H), 4.33 (br, 1H), 4.40 (m, 2H), 5.09 (d, *J* = 2.1 Hz, 2H), 6.90 (d, *J* = 8.4 Hz, 2H), 7.04 (d, *J* = 8.4 Hz, 2H), 7.30 (m, 7H), 7.58 (m, 2H), 7.77 (d, *J* = 7.2 Hz, 2H). 48% Yield.

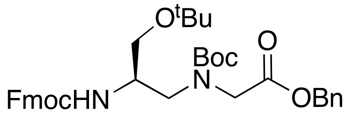

Gly-Ser(^{t}Bu) Monomer Ester: ^{1}H NMR (CDCl$_3$, 300 MHz) δ 1.17 (s, 9H), 1.36 major rotamer/1.47 minor rotamer (s, 9H), 3.35 (m, 2H), 3.55 (m, 2H), 3.92 (m, 2H), 4.13 (q, *J* = 7.2, 14.4 Hz, 2H), 4.22 (m, 1H), 4.32 (m, 2H), 5.18 (s, 2H), 5.75 (d, *J* = 10 Hz, 1H), 7.35 (m, 9H), 7.60 (t, *J* = 7.5 Hz, 2H), 7.76 (d, *J* = 7.8 Hz, 2H). **ESI-MS:** 92% Yield

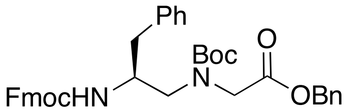

Gly-Phe Monomer Ester: ^{1}H NMR (CDCl$_3$, 300 MHz) δ 1.35 major rotamer/1.40 minor rotamer (s, 9H), 2.90 (m, 2H), 3.16 major rotamer/3.38 minor rotamer (m, 2H), 3.85 (m, 2H), 4.00 (br, 1H), 4.17 (t, *J* = 6.6 Hz, 1H), 4.33 (m, 2H), 5.15 (s, 2H), 5.47 (d, *J* = 6.9 Hz, 1H), 7.34 (m, 8H), 7.40 (t, *J* = 6.9 Hz, 2H), 7.54 (q, *J* = 6.9, 15.6 Hz, 2H), 7.76 (d, *J* = 7.2 Hz, 2H). 75% Yield.

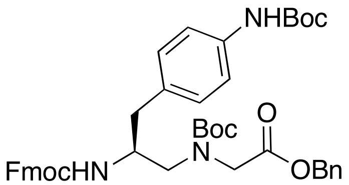

Gly-(4-NH$_2$)Phe(Boc) Monomer Ester: ^{1}H NMR (CDCl$_3$, 300 MHz) δ 1.33 major rotamer/1.40 minor rotamer (s, 9H), 1.51 (s, 9H), 2.77 major rotamer/2.86 minor rotamer (m, 2H), 3.12 major rotamer/ 3.34 minor rotamer (m, 2H), 3.83 (m, 2H), 3.94 (br, 1H), 4.16 (t, *J* = 9 Hz, 1H), 4.31 (m, 2H), 5.14 (s, 2H), 5.48 (d, *J* = 7.2 Hz, 1H), 7.11 (d, *J* = 9 Hz, 2H), 7.32 (m, 9H), 7.38 (t, *J* = 7.2 Hz, 2H), 7.53 (q, *J* = 7, 14 Hz, 2H), 7.74 (d, *J* = 7.2 Hz, 2H). 78% Yield.

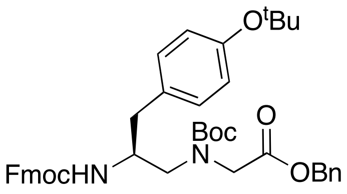

Gly-Tyr(^{t}Bu) Monomer Ester: ^{1}**H NMR** (DMSO, 300 MHz) δ 1.17 minor rotamer/1.19 major rotamer (s, 9H), 1.22 major rotamer/1.31 minor rotamer (s, 9H), 2.62 (m, 2H), 3.08 (m, 1H), 3.44 (m, 1H), 3.85 (br, 1H), 4.13 (m, 5H), 5.15 (d, *J* = 5.1 Hz, 2H), 6.79 (dd, *J* = 1.5, 8.4 Hz, 2H), 7.08 (dd, *J* = 4.0, 5.0 Hz, 2H), 7.19 (dd, *J* = 5.4, 4.4 Hz, 1H), 7.36 (m, 8H), 7.62 (d, *J* = 7.5 Hz, 2H), 7.87 (d, *J* = 7.5 Hz, 2H). 84% Yield.

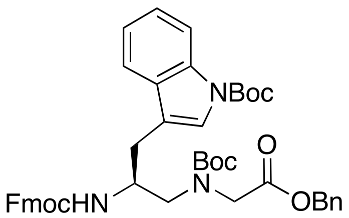

Gly-Trp(Boc) Monomer Ester: ^{1}**H NMR** ($CDCl_3$, 300 MHz) δ 1.35 major rotamer/ 1.38 minor rotamer (s, 9H), 1.65 (s, 9H), 2.90 (m, 1H), 3.14 (m, 1H), 3.80 (m, 2H), 4.15 (m, 2H), 4.33 (m, 2H), 5.13 (d, *J* = 3.6 Hz, 2H), 5.74 (d, *J* = 7.5 Hz, 2H), 7.30 (m, 13H), 7.37 (m, 2H), 7.54 (m, 2H), 7.76 (d, *J* = 8.1 Hz, 1H), 8.12 (d, J = 7.2 Hz, 1H). **ESI-MS:** Expected mass 782.3417, observed mass 782.3389. 75% Yield.

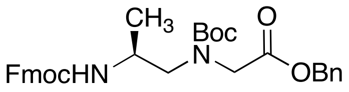

Gly-Ala Monomer Ester: ^{1}**H NMR** ($CDCl_3$, 300 MHz) δ 1.18 (br, 3H), 1.36 major rotamer/ 1.46 minor rotamer (s, 9H), 3.12 major rotamer/3.30 minor rotamer (m, 2H), 3.63 (m, 1H), 3.93 (m, 3H), 4.19 (t, *J* = 6.9 Hz, 1H), 4.35 (m, 2H), 5.17 (s, 2H), 5.45 (br d, 1H), 7.33 (m, 9H), 7.58 (t, *J* = 6.8 Hz, 2H), 7.76 (d, *J* = 7.5 Hz, 2H). 74% Yield.

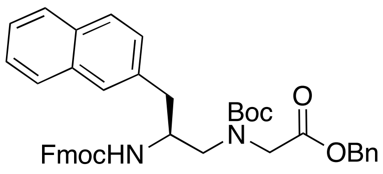

Gly-Nap Monomer Ester: ^{1}**H NMR** ($CDCl_3$, 300 MHz) δ 1.36 major rotamer/ 1.40 minor rotamer (s, 9H), 3.01 (m, 1H), 3.18 (m, 1H), 3.42 (m, 1H), 3.85 (m, 3H), 4.17 (m, 2H), 4.32 (m, 2H), 5.13 (d, *J* = 3.6 Hz, 2H), 5.58 (d, *J* = 7.7 Hz, 1H), 7.23 (m, 2H), 7.32 (m, 6H), 7.38 (t, *J* = 6.6 Hz, 2H), 7.46 (m, 2H), 7.53 (t, *J* = 7.2 Hz, 2H), 7.66 (s, 1H), 7.76 (m, 5H). **ESI-MS:** expected mass 671.3121, observed mass 671.3132. 90% Yield.

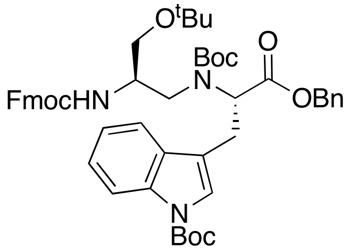

Trp(Boc)-Ser(^{t}Bu) Monomer Ester: ^{1}**H NMR** ($CDCl_3$, 300 MHz) δ 0.99 (s, 9H), 1.40 major rotamer/ 1.45 minor rotamer (s, 9H), 1.64 (s, 9H), 2.76 (m, 1H), 2.93 (br m, 1H), 3.19 (m, 1H), 3.13 (br m, 3H), 3.69 (br, 1H), 4.18 (m, 3H), 4.32 (m, 1H), 5.12 (m, 2H), 5.64 (d, *J* = 5.4 Hz, 1H), 7.26 (m, 10H), 7.38 (t, *J* = 7.2 Hz, 2H), 7.52 (br m, 3H), 7.74 (d, *J* = 7.5 Hz, 2H), 8.12 (br, 1H). **ESI-MS:** expected mass 846.4330, observed mass 846.4323. 77% Yield.

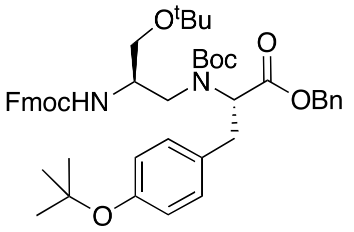

Tyr(^{t}Bu)-Ser(^{t}Bu) Monomer Ester: ^{1}H NMR ($CDCl_3$, 300 MHz) δ 1.06 (s, 9H), 1.32 (s, 9H), 1.44 major rotamer/ 1.45 minor rotamer (s, 9H), 2.54 (br, 1H), 3.14 (m, 1H), 3.34 (m, 4H), 3.68 (br, 1H), 3.95 (br, 1H), 4.20 (m, 2H), 4.35 (m, 2H), 5.16 (m, 2H), 5.65 (d, *J* = 6.6 Hz, 1H), 6.91 (d, *J* = 8.4 Hz, 2H), 7.05 (d, *J* = 6.6 Hz, 2H), 7.28 (m, 7H), 7.40 (t, *J* = 7.8 Hz, 2H), 7.58 (t, *J* = 7.8 Hz, 2H), 7.76 (d, *J* = 7.5 Hz, 2H). 85% Yield.

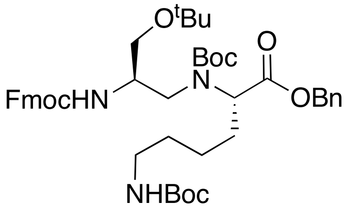

Lys(Boc)-Ser(^{t}Bu) Monomer Ester: ^{1}H NMR ($CDCl_3$, 300 MHz) δ 1.14 (s, 9H), 1.36 (s, 9H), 1.42 (s, 9H), 1.50 (m, 5H), 1.93 (m, 1H), 3.10 (br, 2H), 3.25 (m, 2H), 3.50 (m, 1H), 3.82 (m, 1H), 4.04 (br, 1H), 4.21 (m, 1H), 4.32 (m, 2H), 4.67 (br, 1H), 5.12 (s, 2H), 5.51 (br, 1H), 5.92 (br, 1H), 7.32 (br m, 7H), 7.39 (t, *J* = 7.5 Hz, 2H), 7.60 (d, *J* = 6.9 Hz, 2H), 7.76 (d, *J* = 7.5 Hz, 2H). 70% Yield.

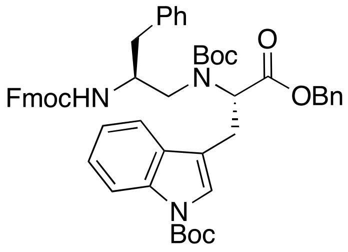

Trp(Boc)-Phe Monomer Ester: ^{1}H NMR ($CDCl_3$, 300 MHz) δ 1.41 (s, 9H), 1.65 (s, 9H), 2.57 (m, 2H), 3.29 (m, 2H), 3.49 (m, 2H), 3.79 (br, 1H), 4.11 (m, 3H), 4.31 (br, 1H), 5.09 (m, 2H), 5.69 (d, *J* = 1.6 Hz, 1H), 6.76 (br, 1H), 6.86 (br, 1H), 7.27 (m, 18H), 7.75 (d, *J* = 7.69 Hz, 2H), 8.11 (br, 1H). 81% Yield.

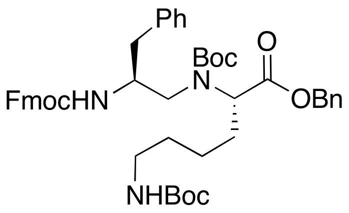

Lys(Boc)-Phe Monomer Ester: ^{1}H NMR ($CDCl_3$, 300 MHz) δ 1.36 (s, 9H), 1.43 (s, 9H), 1.4 (m, 3H), 1.78 (m, 3H), 2.76 (m, 1H), 2.93 (m, 1H), 3.05 (m, 2H), 3.90 (m, 2H), 4.19 (m, 2H), 4.36 (m, 2H), 4.61 (br, 1H), 5.09 (m, 2H), 5.51 (br, 1H), 5.89 (br, 1H), 7.03 (br d, *J* = 6.2 Hz, 1H), 7.11 (br d, *J* = 6.9 Hz, 1H), 7.29 (m, 10H), 7.40 (t, *J* = 7.2 Hz, 2H), 7.56 (t, *J* = 7.5 Hz, 2H), 7.76 (d, *J* = 6.9 Hz, 2H). 68% Yield.

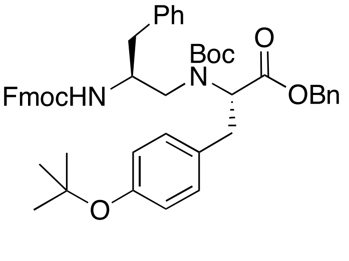

Tyr(^{t}Bu)-Phe Monomer Ester: ^{1}H NMR ($CDCl_3$, 300 MHz) δ 1.33 (s, 9H), 1.41 (s, 9H), 2.18 (d, J = X Hz, 1H), 2.37 (d, J = X Hz, 1H), 2.57 (m, 1H), 2.79 (m, 1H), 3.16 (m, 2H), 3.47 (m, 1H), 3.74 (m, 1H), 4.12 (m, 1H), 4.28 (m, 2H), 5.07 (m, 2H), 5.69 (d, *J* = 6.6 Hz, 1H), 6.87 (m, 2H), 6.95 (d, *J* = 6.6 Hz, 2H), 7.28 (m, 12H), 7.40 (t, *J* = 6.9 Hz, 2H), 7.49 (d, *J* = 6.7 Hz, 1H), 7.56 (d, *J* = 6.7 Hz, 1H), 7.77 (d, *J* = 6.6 Hz, 2H). 91% Yield.

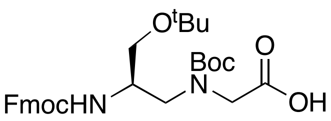

Gly-Ser(tBu) Monomer: ^{1}**H NMR** (DMSO, 300 MHz) δ 1.11 (s, 9H), 1.33 major rotamer/ 1.38 minor rotamer (s, 9H), 3.02 (m, 2H), 3.32 (m, 2H), 3.72 (br, 1H), 3.84 (m, 2H), 4.26 (m, 3H), 7.04 (d, *J* = 8.6 Hz, 1H), 7.33 (t, *J* = 6.9 Hz, 2H), 7.41 (t, *J* = 7.7 Hz, 2H), 7.65 (d, *J* = 6.8 Hz, 2H), 7.88 (d, *J* = 8.1 Hz, 2H). **ESI-MS:** expected mass 525.2542, observed mass 525.2564. 88% Yield.

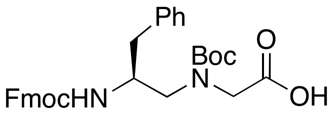

Gly-Phe Monomer: ^{1}**H NMR** (DMSO, 300 MHz) δ 1.32 (s, 9H), 2.70 (m, 3H), 3.04 (m, 1H), 3.41 (m, 1H), 3.84 (m, 2H), 4.17 (m, 3H), 7.22 (m, 7H), 7.32 (t, *J* = 7.2 Hz, 2H), 7.41 (t, *J* = 7.5 Hz, 2H), 7.61 (m, 2H), 7.88 (d, *J* = 7.5 Hz, 2H). **ESI-MS:** expected mass 529.2339, observed mass 529.2341. 83% Yield.

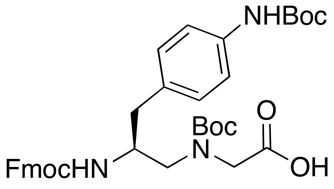

Gly-(4-NH_2)Phe Monomer: ^{1}**H NMR** ($CDCl_3$, 300 MHz) δ 1.40 (s, 9H), 1.50 (s, 9H), 2.80 (m, 2H), 3.08 (d, J = X Hz, 1H), 3.34 (m, 1H), 3.77 (m, 2H), 3.93 (br, 1H), 4.14 (t, *J* = 6.3 Hz, 1H), 4.29 (m, 2H), 5.55 (br d, 2H), 7.09 (br d, 2H), 7.27 (m, 4H), 7.34 (t, *J* = 7.5 Hz, 2H), 7.51 (d, *J* = 6.9 Hz, 2H), 7.73 (d, *J* = 7.2 Hz, 2H). **ESI-MS:** expected mass 646.3128, observed mass 646.3135. 58% Yield.

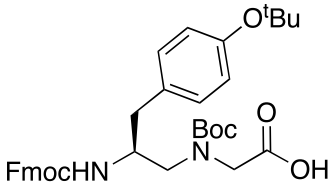

Gly-Tyr(^{t}Bu) Monomer: ^{1}**H NMR** (DMSO, 300 MHz) δ 1.18 major rotamer/1.20 minor rotamer (s, 9H), 1.32 (s, 9H), 2.60 (m, 2H), 3.04 (m, 1H), 3.41(m, 1H), 3.85 (m, 3H), 4.13 (m, 3H), 6.78 (dd, *J* = 1.2, 8.7 Hz, 2H), 7.10 (dd, *J* = 4.2, 3.9 Hz, 2H), 7.32 (t, *J* = 7.2 Hz, 2H), 7.41 (t, *J* = 7.2 Hz, 2H), 7.62 (d, *J* = 5.4 Hz, 2H), 7.88 (d, *J* = 7.5 Hz, 2H). ESI-MS: expected mass 601.2914, observed mass 601.2885. 85% Yield.

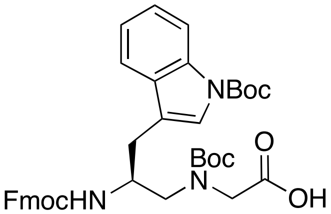

Gly-Trp(Boc) Monomer: ^{1}**H NMR** (DMSO, 300 MHz) δ 1.21 major rotamer/1.33 minor rotamer (s, 9H), 1.55 (s, 9H), 2.78 (m, 2H), 3.13 (m, 1H), 3.48 (m, 1H), 3.90 (m, 3H), 4.18 (m, 3H), 7.36 (m, 7H), 7.51 (m, 2H), 7.67 (t, *J* = 6.7 Hz, 2H), 7.87 (d, *J* = 7.8 Hz, 2H), 8.02 (d, *J* = 8.1 Hz, 1H). 81% Yield.

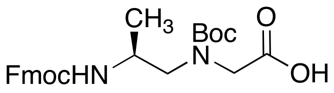

Gly-Ala Monomer: ^{1}**H NMR** (DMSO, 300 MHz) δ 1.01 (d, *J* = 5.4 Hz, 3H), 1.33 major rotamer/ 1.38 minor rotamer (s, 9H), 3.04 (m, 1H), 3.27 (m, 1H), 3.69 (m, 1H), 3.83 (m, 2H), 4.24 (m, 3H), 7.32 (t, *J* = 7.2 Hz, 2H), 7.41 (t, *J* = 7.8 Hz, 2H), 7.68 (d, *J* = 7.5 Hz, 2H), 7.89 (d, *J* = 7.2 Hz, 2H). ESI-MS: expected mass 453.2026, observed mass 453.2007. 93% Yield.

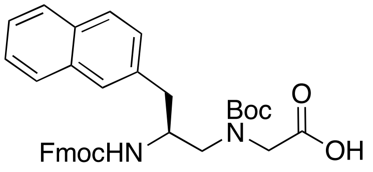

Gly-Nap Monomer: ^{1}H NMR ($CDCl_3$, 300 MHz) δ 1.40 (s, 9H), 3.07 (m, 3H), 3.41 (m, 1H), 3.73 (m, 1H), 3.86 (d, *J* = 3.0 Hz, 1H), 3.95 (br d, 1H), 4.13 (q, *J* = 7.2, 14.4 Hz, 1H), 4.34 (m, 2H), 5.57 (br d, 1H), 7.22 (m, 2H), 7.37 (m, 3H), 7.45 (m, 4H), 7.65 (m, 1H), 7.75 (m, 5H). **ESI-MS:** expected mass 579.2495, observed mass 579.2507. 92% Yield.

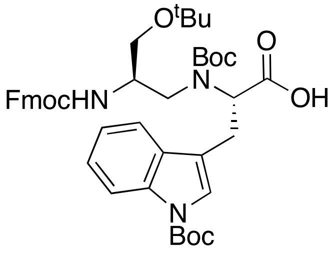

Trp(Boc)-Ser(^{t}Bu) Monomer: ^{1}H NMR ($CDCl_3$, 300 MHz) δ 1.00 major rotamer/ 1.05 minor rotamer (s, 9H), 1.47 (s, 9H), 1.66 (s, 9H), 3.21 (m, 4H), 3.53 (m, 2H), 3.71 (br, 1H), 4.06 (m, 1H), 4.17 (t, *J* = 6.9 Hz, 1H), 4.31 (d, *J* = 6.3 Hz, 2H), 5.51 (d, *J* = 8.1 Hz, 1H), 7.29 (m, 4H), 7.39 (m, 3H), 7.55 (m, 3H), 7.75 (d, *J* = 7.5 Hz, 2H), 8.13 (d, *J* = 7.2 Hz, 1H). **ESI-MS:** expected mass 754.3704, observed mass 754.3698. 88% Yield.

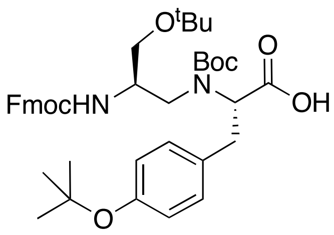

Tyr(^{t}Bu)-Ser(^{t}Bu) Monomer: ^{1}H NMR ($CDCl_3$, 300 MHz) δ 1.10 (s, 9H), 1.32 (s, 9H), 1.46 (s, 9H), 2.12 (br d, 1H), 3.27 (m, 4H), 3.74 (m, 2H), 3.96 (m, 1H), 4.19 (t, *J* = 7.2 Hz, 1H), 4.33 (d, *J* = 6.6 Hz, 2H), 5.50 (d, *J* = 7.8 Hz, 1H), 6.92 (d, *J* = 8.4 Hz, 2H), 7.03 (d, *J* = 8.1 Hz, 2H), 7.30 (t, *J* = 7.5 Hz, 2H), 7.39 (m, 2H), 7.58 (t, *J* = 6.6 Hz, 2H), 7.75 (d, *J* = 7.5 Hz, 2H). **ESI-MS:** expected mass 687.3645, observed mass 687.3649. 66% yield.

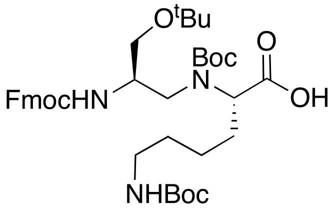

Lys(Boc)-Ser(^{t}Bu) Monomer: ^{1}H NMR ($CDCl_3$, 300 MHz) δ 1.18 (s, 9H), 1.42 (s, 22H), 1.80 (br, 1H), 2.04 (br, 1H), 3.09 (m, 3H), 3.45 (m, 3H), 3.91 (m, 2H), 4.20 (m, 1H), 4.33 (d, *J* = 6.0 Hz, 2H), 5.48 (br, 1H), 5.84 (br, 1H), 7.30 (t, *J* = 7.5 Hz, 2H), 7.39 (t, *J* = 7.2 Hz, 2H), 7.59 (d, *J* = 7.2 Hz, 2H), 7.75 (d, *J* = 7.8 Hz, 2H). **ESI-MS:** expected mass 696.3860, observed mass 696.3864. 73% Yield.

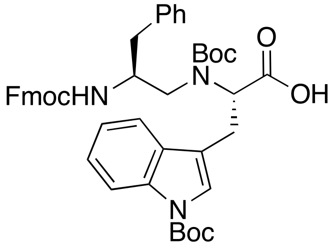

Trp(Boc)-Phe Monomer: ^{1}H NMR ($CDCl_3$, 300 MHz) δ 1.42 (s, 9H), 1.66 (s, 9H), 2.20 (br, 1H), 2.53 (m, 2H), 3.33 (m, 3H), 3.85 (br, 2H), 4.11 (br, 1H), 4.28 (br, 2H), 5.42 (br, 1H), 6.89 (br, 2H), 7.15 (m, 5H), 7.32 (m, 7H), 7.48 (br, 2H), 7.70 (br, 2H), 8.12 (d, *J* = 7.5 Hz, 2H). **ESI-MS:** expected mass 758.3441, observed mass 758.3440. 81% Yield.

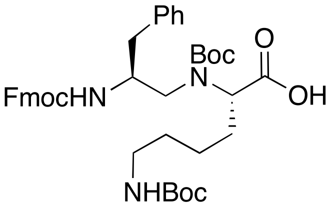

Lys(Boc)-Phe Monomer: ^{1}H NMR ($CDCl_3$, 300 MHz) δ 1.38 major rotamer/ 1.35 minor rotamer (s, 9H), 1.34 (m, 2H), 1.42 (s, 9H), 1.63 (m, 2H), 1.90 (m, 2H), 3.02 (m, 4H), 3.48 (br, 1H), 3.77 (m, 1H), 4.02 (br, 1H), 4.15 (br, 1H), 4.32 (m, 2H), 4.59 (br, 1H), 5.85 (br, 1H), 7.27 (m, 7H), 7.37 (t, *J* = 7.5 Hz, 2H), 7.53 (d, *J* = 7.2 Hz, 2H), 7.73 (d, *J* = 7.2 Hz, 2H). **ESI-MS:** 700.3598, observed mass 700.3615. 67% Yield.

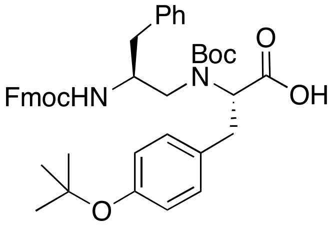

Tyr(^{t}Bu)-Phe Monomer: ^{1}H NMR ($CDCl_3$, 300 MHz) δ 1.33 major rotamer/ 1.32 minor rotamer (s, 9H), 1.42 major rotamer/ 1.38 minor rotamer (s, 9H), 2.47 (m, 1H), 2.76 (m, 1H), 3.03 (m, 1H), 3.16 (m, 2H), 3.41 (m, 1H), 3.57 (m, 1H), 3.84 (m, 1H), 4.13 (t, *J* = 6.3 Hz, 1H), 4.30 (d, *J* = 7.8 Hz, 2H), 6.83 (s, 2H), 7.03 (br, 2H), 7.24 (m, 7H), 7.36 (q, *J* = 5.4, 12.6 Hz, 2H), 7.50 (d, *J* = 7.5 Hz, 2H), 7.72 (dd, *J* = 3.6, 3.3 Hz, 2H). **ESI-MS:** expected mass 691.3383, observed mass 691.3363. 80% Yield.

Selected NMRs of PAA Monomers:

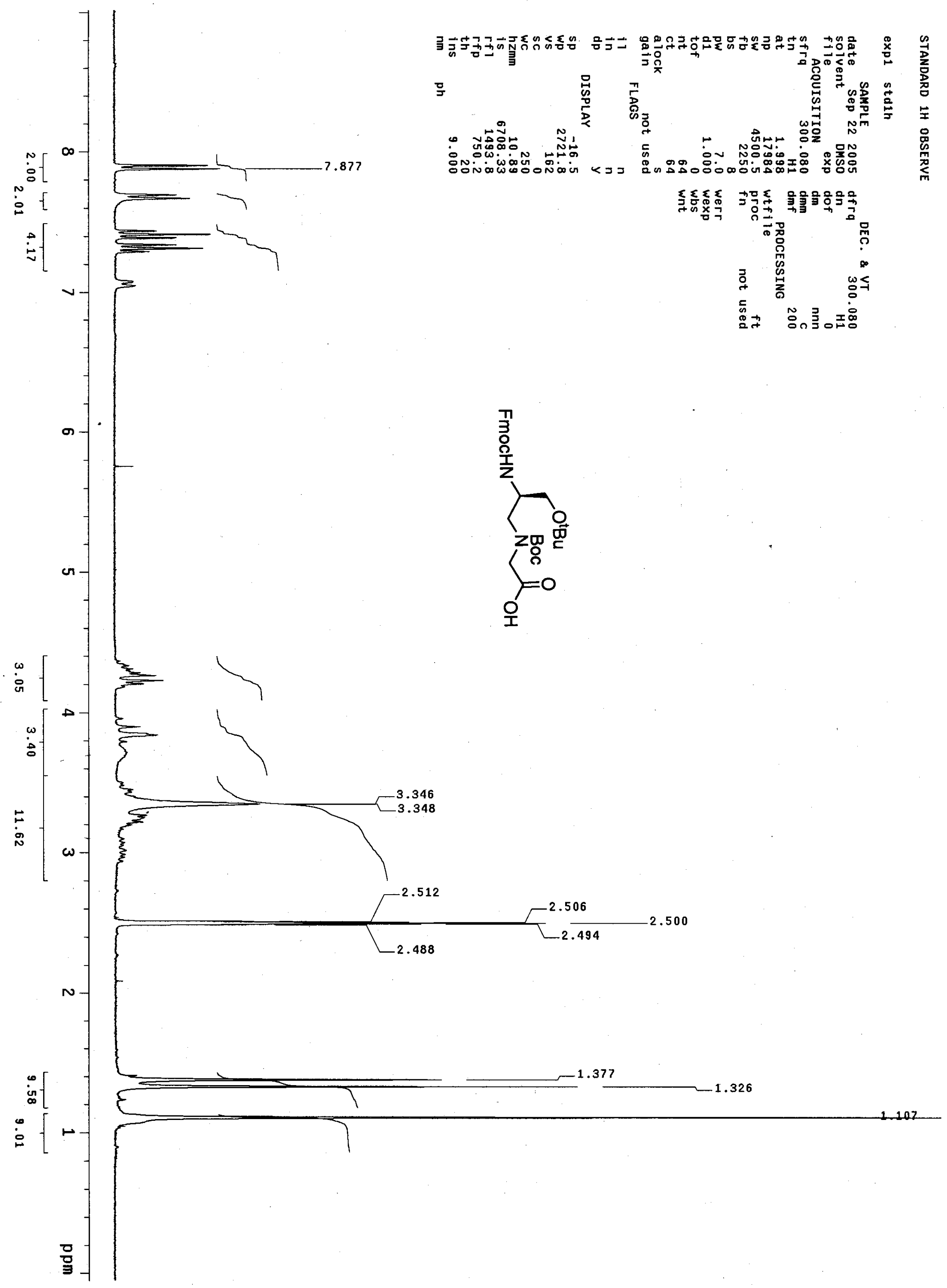

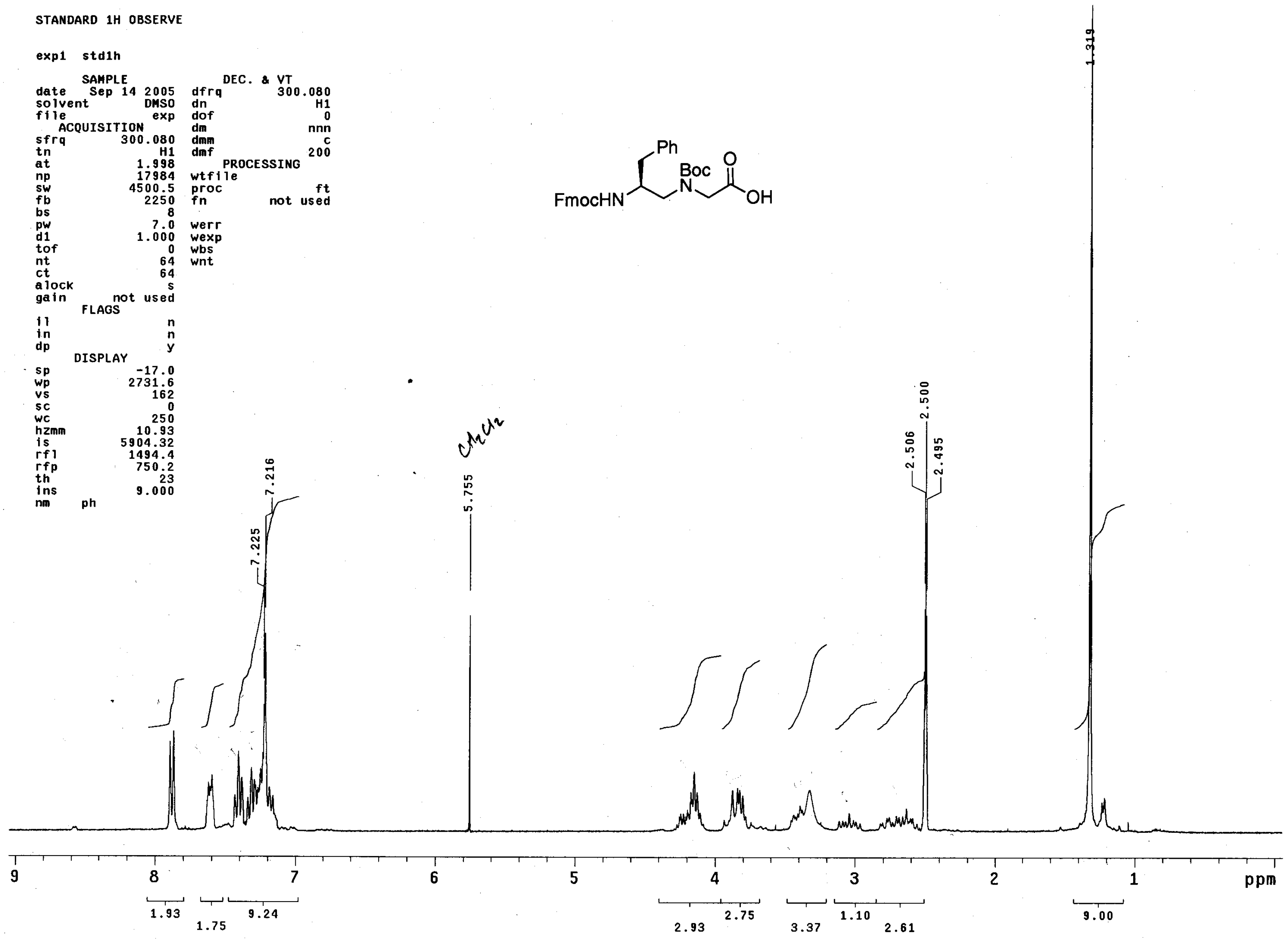
STANDARD 1H OBSERVE
exp1 std1h
SAMPLE
date Sep 14 2005
solvent DMSO
file exp
ACQUISITION
sfrq 300.080
tn H1
at 1.998
np 17984
sw 4500.5
fb 2250
bs 8
pw 7.0
d1 1.000
tof 0
nt 64
ct 64
alock s
gain not used
FLAGS
il n
in n
dp y
DISPLAY
sp -17.0
wp 2731.6
vs 162
sc 0
wc 250
hzmm 10.93
is 5904.32
rfl 1494.4
rfp 750.2
th 23
ins 9.000
nm ph
DEC. & VT
dfrq 300.080
dn H1
dof 0
dm nnn
dmm c
dmf 200
PROCESSING
wtfile
proc ft
fn not used
werr
wexp
wbs
wnt
Ph
Boc
O
FmocHN
N
OH
7.225
7.216
5.755
2.506
2.500
2.495
1.319
9
8
7
6
5
4
3
2
1
ppm
1.93
1.75
9.24
2.93
2.75
3.37
1.10
2.61
9.00

General Procedure F: Solid Phase Synthesis of PAA Trimers

Note on resin washes: All washes used at least enough solution to cover the resin (~1.5 mL for 100 mg resin, and ~5 mL for 1 g resin). Values in parentheses describe the number of times the resin is washed with the indicated solution, and the agitation time for each wash. For example, (4x-30s) indicates four washes, with each having an agitation time of 30 seconds and each followed by draining under vacuum. If no time or number of repetitions is indicated, a single wash and/or a five second agitation period is used.

1. Downloading Resin
Rink resin (1.0 g, 0.75 mmol active sites/gram) is downloaded to 0.1 mmol/g with Fmoc-β-alanine-COOH. The resin is first swelled in DCM for between 1 and 12 hours. The following solutions are prepared: 0.2 M Fmoc-β-alanine-COOH in NMP (A), 0.2 M HATU in NMP (B), and 0.5 M DIEA in NMP (C). These solutions are then combined appropriately to give two additional solutions: 0.45 mL of A + 0.46 mL of C + 1.59 mL NMP (Solution 1), and 0.55 mL of B + 1.95 mL NMP (Solution 2). Solutions 1 and 2 are pre-mixed for one minute and then added to the resin. The resin is agitated with N_2 bubbling for one hour and then drained under vacuum. The resin is subsequently washed with DMF (2x), DCM (4x), 5% DIEA in DCM (1x-30s), and again with DCM (4x). The remaining active sites are then capped with a 1:2:2 solution of Ac_2O:NMP:pyridine for 1.5 hours. This is followed by washes with DCM (2x-5s) and a qualitative Kaiser test to confirm that no primary amines remain. Resin is then washed with DCM (5x) and allowed to dry under vacuum for 30-60 minutes. Downloaded resin is stored in a dessicator until further use.

2. PAA Synthesis
The following is a representative coupling cycle for one PAA monomer. Downloaded resin (100 mg) is swelled in DCM for 1hr. The solvent is drained under vacuum and a solution of 20% piperidine in DMF is added to the resin. The resin is agitated for 6 minutes, and the solution is removed under vacuum. The Fmoc deprotection is repeated three times to ensure complete deprotection. This is followed by subsequent washes with DCM, DMF, DCM (2x-5s), and pyridine (2x-5s). A Kaiser test is performed to confirm deprotection. Upon positive Kaiser test, 300 μL of a 0.2 M solution of PAA monomer in NMP is pre-mixed with 300 μL of 0.8 M DIEA in pyridine and 300 μL of 0.2 M HATU in DMF for one minute. This solution is then added to the resin and agitated for 60 minutes. Following coupling, the resin was drained under vacuum and washed with DMF (1x-5s, 1x-30s, 1x-5s), 5% DIEA/DCM (1x-30s) and DCM (2x-5s). Again, a qualitative Kaiser test is performed and, if negative, the resin is capped with a 1:25:25 mixture of Ac_2O: NMP: pyridine (2x-2min). (Note: If the Kaiser test is positive, the coupling cycle is repeated, beginning with the pyridine washes.) The capping step is followed by washes with DCM (1x-5s, 1x-30s, 1x-5s). This cycle is then repeated iteratively until the oligomer is complete on resin.

3. Cleavage of PAA from Resin
Cleavage from the resin is accomplished under acidic conditions. After the final Fmoc-deprotection, the resin is washed with CH_2Cl_2, DMF, CH_2Cl_2 (2x) and drained under vacuum. Next, 1000 μL of a solution consisting of 50 μL m-cresol, 200 μl TFA, and 750 μL CH_2Cl_2 is added to the resin and agitated for 20 minutes. The resulting solution is collected using positive

N_2 pressure to force liquid through the fritted vessel. The cleavage solution is evaporated by blowing dry N_2 over the crude product. Following evaporation, the crude product is re-dissolved in 1 mL of a 95/5 solution of TFA/m-cresol and allowed to sit for 2 hours to deprotect the backbone and sidechains. The resulting crude product again has a stream of N_2 passed over the reaction vessel to remove volatiles.

4. Crude PAA Isolation
The resulting oil is partitioned between five 2.0 mL microcentrifuge tubes using a micropipeter, to typically yield 100-150 μL of the oil per tube. Each tube is filled to capacity with diethyl ether. The solutions are mixed by vortexing until the brown color no longer remains and a cloudy white precipitate forms. The solutions are then placed on dry ice for ten minutes. This is followed by centrifugation (5 min at 7000 rpm) and removal of solvent via decanting or pipeting to yield a white solid as the crude PNA product. The ether precipitation cycle is repeated twice.. After decanting the final ether wash, residual solvent is removed by passing a stream of N_2 over the crude PAA product.

Library Synthesis

Tentagel-NH_2 macrobeads (0.2 mmol/g, ~300 μm/bead) are partitioned between 125 wells (~5 mg/well) in two 96-well plates. Each well is swelled in DMF (100 μL/well) for 30 minutes prior to solid phase synthesis. The synthesis of PAA trimers proceeds as described above with the following changes. Resin was agitated using a mechanical shaker rather than N_2 bubbling. All resin washes and deprotections were carried out using 100 μL of the indicated solution. Also, Tentagel macrobeads were used at the given loaded capacity and were not further downloaded. All couplings (including β-alanine) were done with a 12-fold excess of monomer and coupling agent to ensure complete coupling (30 μL 0.4M PAA monomer + 60 μL 0.2 M HATU + 30 μL 0.8 M DIEA). After the PAA synthesis was complete, each well was subjected to 95:5 ratio of TFA:m-cresol for 2 hours to deprotect the sidechain and the backbone. The acidic solution was then drained under vacuum and the resin was washed several times with CH_2Cl_2 (at least 5x) and stored in a dessicator for further use.

Mass Spectral Data for PAA Trimers

Sequence	Expected Mass	Observed Mass
SFF	599.3669	599.3658
FFF	659.4	659.4
YFF	675.4	675.4
SYS	555.3	555.3
FYY	691.4	691.4
YSF	615.4	615.3
AFF	583.4	583.3
SAF	523.3	523.3
SFA	523.3	523.3
KSGFYF	776.5	776.4
WSGFKF	799.5	799.4
GSYFKF	776.5	776.4
KSYFGF	776.5	776.4
KSKFKF	812.6	812.5
YSKFKF	847.5	847.5

High-throughput Screen for TAR RNA Binding

1. Transcription of Single Base Bulge Mutant TAR

Transcription of the mutant TAR RNA was carried out using protocols outlined in the MEGAscriptT7 transcription kit (Ambion). Briefly, double stranded TAR mutant DNA (Sense: 5'- GGC CAG AGA GCT CCC AGG CTC GTC TGG CCT ATA GTG AGT CGT ATT A- 3', Antisense: 5'-TAA TAC GAC TCA CTA TAG GCC AGA CGA GCC TGG GAG CTC TCT GGC C- 3') was dissolved in water to a concentration of 1 μg/μL. The transcription reagents were then thawed on ice, but the following transcription reaction was carried out at room temperature. Combined in a centrifuge tube were: 21 μL Nuclease Free H_2O, 6 μL ATP Solution, 6 μL CTP solution, 6 μL GTP solution, 6 μL UTP solution, 6 μL 10x reaction buffer, 3 μL template DNA, and 6 μL enzyme mix. The reaction was incubated at 37 ^{o}C for 16 hours, after which 3 μL of TurboDNAse was added. The mixture was incubated at 37 ^{o}C for an additional 15 minutes. Ethanol was added (150 μL) and RNA was allowed to precipitate in the freezer (2 hours, -20 ^{o}C). The mixture was centrifuged on a microcentrifuge at the maximum speed for 30 minutes and the ethanol was decanted. The resulting RNA pellet was redissolved in water to yield the TAR mutant (5'- GGC CAG ACG AGC CUG GGA GCU CUC UGG CC- 3').

2. Screen for TAR RNA binding to PAA Library Members

Several beads from each well of the PAA library were transferred to a 384-well filter plate, keeping their spatial separation and orientation intact. The beads were first washed with water (5x – 50 μL), then 1x TK buffer (50 mM Tris, 20 mM KCl, 0.1% Triton X-100, pH 7.4; 4x – 50 μL). To each well, BSA (0.1 mg/mL) was added in 1x TK buffer (20 μL) and agitated with

mechanical shaking for 60 minutes at room temperature. The microplate was drained under vacuum and washed with 1x TK buffer (3x – 50 μL). Following BSA blocking, bulge mutant TAR in 1x TK buffer (2.5 μM, 20 μL/well) was added to each individual well. The library was incubated with bulge mutant TAR for 24 hours at 4 ^{o}C before being drained under vacuum. Immediately following solvent removal, a mixture of bulge mutant TAR (2.5 μM) and 5'-biotin labeled TAR (Dharmacon, 250 nM) in 1x TK buffer (20 μL/ well) were introduced to the library. (Note: RNA was snap-cooled by heating at 95 ^{o}C for 5 minutes followed by an immediate transfer to dry ice for 5 minutes to promote hairpin formation) The library was incubated with this solution for 2.5 days at 4 ^{o}C, drained, and washed with water. To each well was added a solution of Qdot605 (50 nM, 15 μL/well) in 1x TK buffer that was agitated at room temperature for 3 hours. The solution was drained and each well was washed with 1x TK (3x – 50 μL), followed by a 2 hour wash with 1x TK buffer and drainage under vacuum. The library was then visualized using a fluorescent microscope equipped with a triple bandpass filter. Beads that appeared red or orange under the microscope were selected for further characterization while those that were green were disregarded.

Terbium Footprinting Assays

1. Transcription of TAR RNA

Transcription of TAR RNA for footprinting studies was carried out as described for the bulge mutant TAR in the screening assay. The sole difference was the DNA template used for transcription, which follows: TAR (-) 5'- GGC CAG AGA GCT CCC AGG CTC AGA TCT GGC CTA TAG TGA GTC GTA TTA- 3', TAR (+) 5'- TAA TAC GAC TCA CTA TAG GCC AGA TCT GAG CCT GGG AGC TCT CTG GCC- 3'.

2. 5'- Radiolabeling of TAR RNA

All RNA dephosphorylation and kinasing reactions were carried out based on protocols provided with the KinaseMax kit (Ambion). The dephosphorylation was assembled as follows; 1.0 μL, TAR RNA (4 μM stock), 2.0 μL 10x dephosphorylation buffer, 2.0 μL calf intestine alkaline phosphatase, 15 μL nuclease free water, and then incubated at 37 ^{o}C for 60 minutes. Then, phosphatase removal reagent (PRR) was added (20 μL) and the mixture was stirred at room temperature for 3 minutes. The reaction tube was briefly pulsed in a microcentrifuge and the supernatant was collected for the kinasing reaction (~ 30 μL). To the supernatant, was added 2.0 μL ^{32}P-ATP (6000 mCi/mL), 4.0 μL 10x Kinase buffer, and 2.0 μL T4 Kinase. The reaction was incubated for 1 hour at 37 ^{o}C and then sent through NucAway spin columns following the instructions provided by the manufacturer (Ambion).

3. Terbium Footprinting Assay

The terbium footprinting assay is essentially a titration of PAA ligand into a system containing a constant amount of ^{32}P-labeled TAR RNA. The concentration of PAA was varied between 0 μM and 1000 μM for all PAAs studied. The assay conditions in each individual tube otherwise stayed constant and were set to meet the following specifications; 50 mM Tris, 10 mM $MgCl_2$, 100 mM NaCl, 3 mM $TbCl_3$, pH 6.5, ~200 fmol ^{32}P-TAR and Δ [PAA]. (Note: RNA was snap-cooled by heating at 95 ^{o}C for 5 minutes followed by an immediate transfer to dry ice for 5 minutes to promote hairpin formation) Thirteen reactions were typically assembled to have a

total volume of 10 μL and the following PAA concentrations (μM): 0, 0.2, 0.4, 1, 2, 4, 10, 20, 40, 100, 200, 400, 1000. The reaction mixtures were incubated at room temperature for 4 hours and were consequently quenched with 10 μL gel loading buffer (Ambion).

4. Gel Electrophoresis

Prior to electrophoretic analysis of the Tb cleavage assays, gel plates were meticulously washed and rinsed with deionized water. The plates were then silanized following manufacturers protocols (Sigmacote, Aldrich) and again meticulously washed and rinsed. The plates were rinsed once more with absolute ethanol and dried with a Kimwipe to prepare them for use. A 15% denaturing polyacrylamide gel solution was prepared (19.2g Urea, 15 mL 40% acrylamide/bisacrylamide Solution (19:1), 7 mL H_2O, 4 mL 10x TBE). To the acrylamide solution was added a 10% weight percent aqueous solution of ammonium persulfate (400 μL) and N,N,N',N'-Tetramethylethylenediamine (40 μL). The gel solution was quickly poured into sequencing gel plates separated by 0.4 mm spacers and held in place with a casting clamp (LabRepco). While still liquid, a 20-well sequencing comb was inserted at the top of the gel and the gel was secured with binder clips. The gel was allowed to set for between 1 and 2 hours. Following polymerization, excess acrylamide was removed from the exterior surface of the plates and the comb was removed.

The newly cast gel was fastened into a sequencing apparatus and the gel was pre-run (40W, 20 minutes) with 1x TBE occupying both upper and lower buffer chambers. While the gel was pre-running two control cleavage reactions were assembled as a means to identify nucleotides in the Tb footprint assays. A base-catalyzed hydrolysis was used to cleave all nucleotides and give a picture of all bases in the TAR hairpin. The reaction contained 3 μL RNA (~600 fmol) and 7 μL alkaline hydrolysis buffer and was heated to 95 ^{o}C for 10 minutes before quenching with 10 μL gel loading buffer. The alternative control was a T1 nuclease mediated cleavage, cleaving only guanine-containing positions of the TAR hairpin. The reaction contained 2 μL TAR (~400 fmol), 7 μL sequencing buffer, 0.3 μL Yeast RNA, 1 μL T1 nuclease and was incubated for 15 minutes at room temperature before quenching with 10 μL gel sequencing buffer.

Following the gel pre-run, all wells were thoroughly washed with 1x TBE buffer to remove excess acrylamide and precipitated urea. All samples (PAA footprinting and controls) were heated to 95 ^{o}C in a dry bath for 2 minutes prior to loading onto the gel. 4 μL of each sample was loaded into each individual well on the sequencing gel using a micropipetter equipped with gel loading tips. Once all samples were loaded, the gel was run at 40W for 75 minutes. The gel plates were then removed from the sequencing apparatus and cooled to room temperature under a stream of cold water. The gel plates were placed flat on a benchtop and separated using a straight edge razor blade, leaving the gel face-up on the bottom plate. Two pieces of sequencing paper were placed on top of the gel and wetted with water using a squirt bottle. The entire assembly was inverted and the gel/sequencing paper was separated from the glass plate. The gel was then dried in a gel dryer for 1 hour at 80 ^{o}C. The dried gel was placed in an imaging cassette with a phosphorimaging screen overnight. The screen was removed and analyzed using a phosphorimager to produce an image of the gel. The image was then quantified using ImageQuant software and intensities were plotted using Prism to derive binding curves.

RNA Footprinting Gels

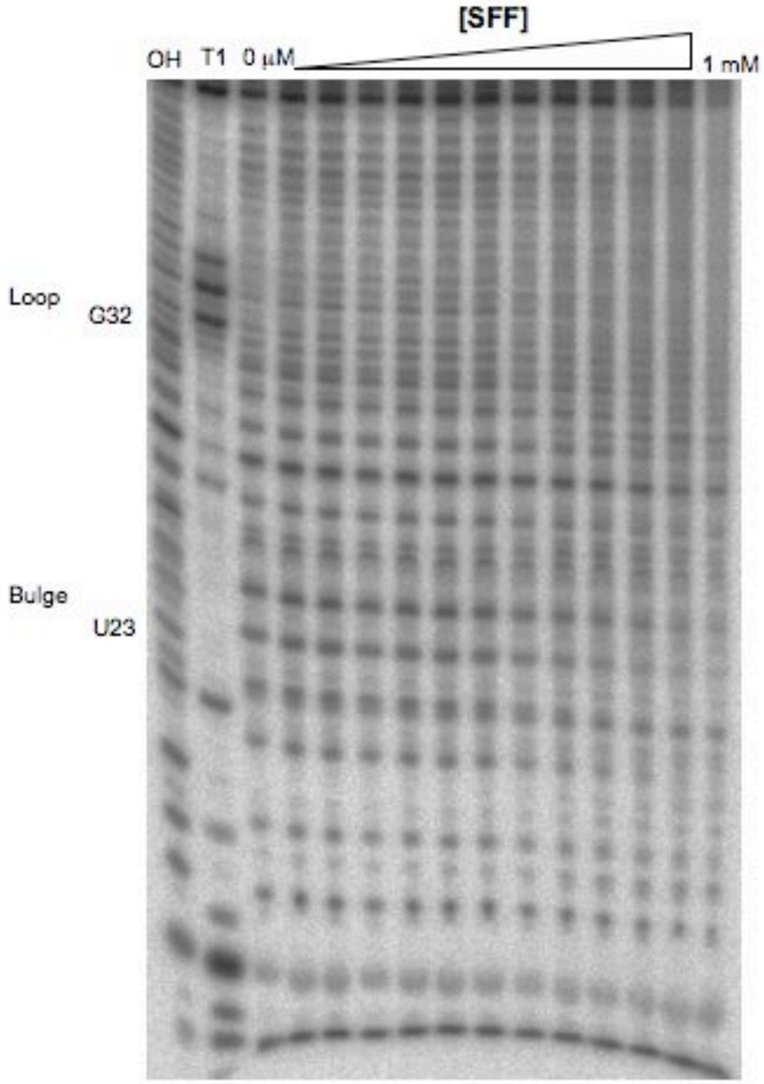

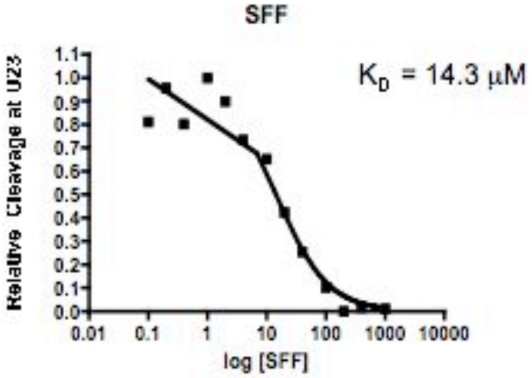

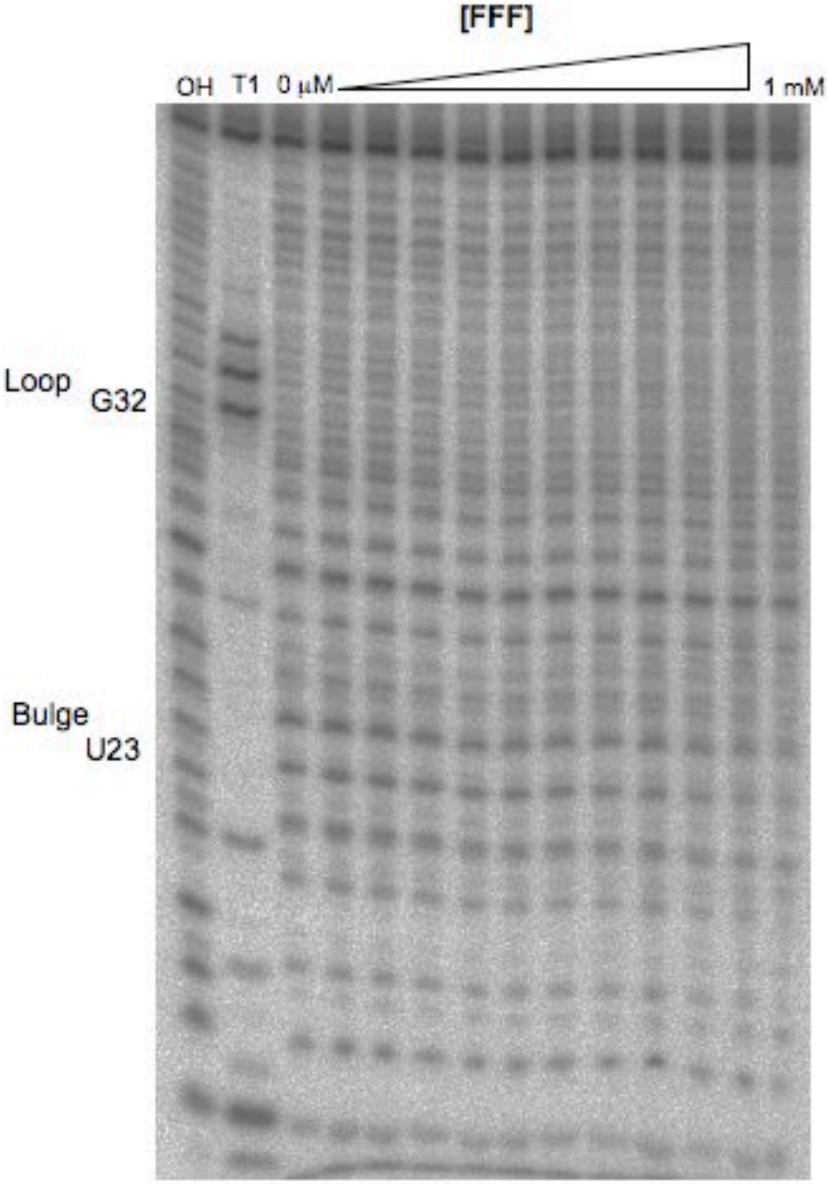
[FFF]
OH
T1
0 μM
1 mM
Loop
G32
Bulge
U23

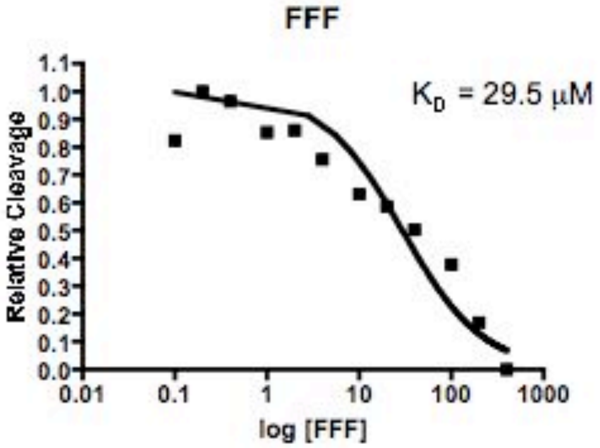
FFF
K_D = 29.5 μM
Relative Cleavage
log [FFF]
0.01
0.1
1
10
100
1000

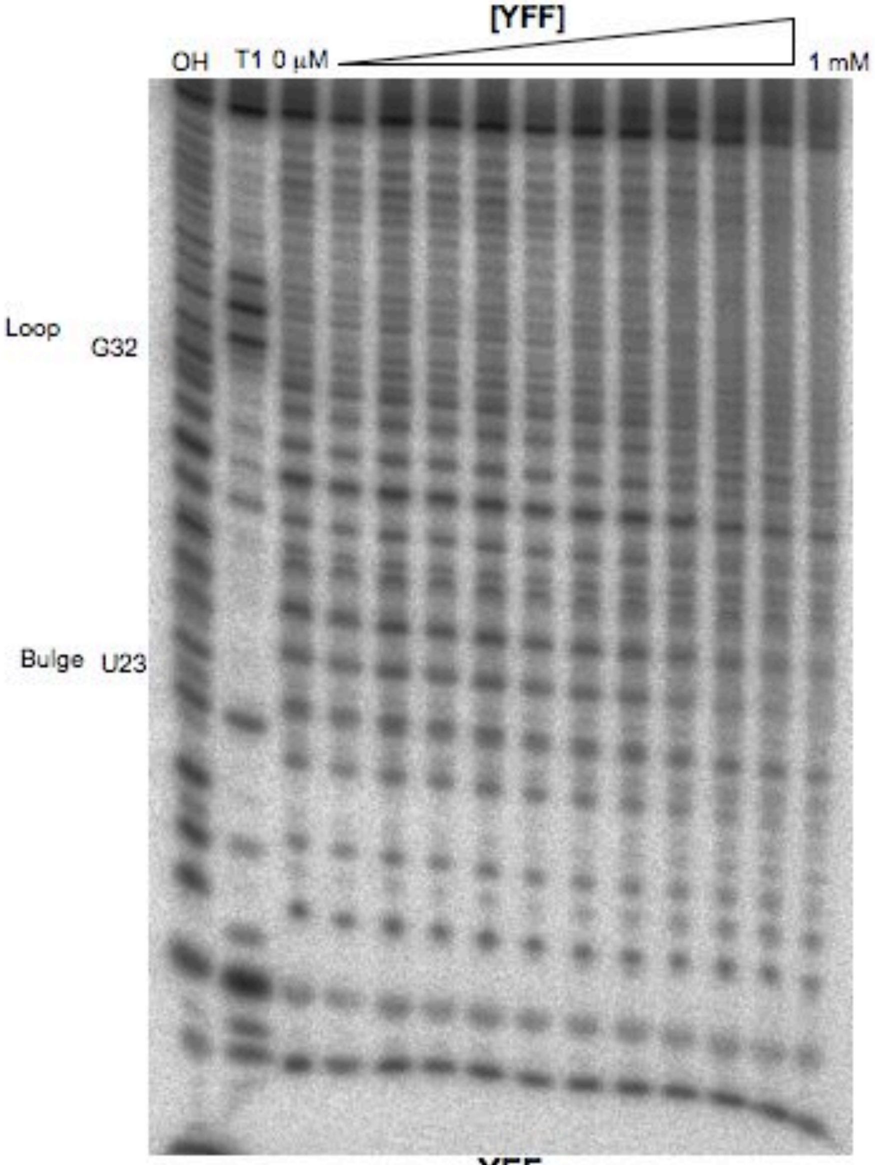
[YFF]
OH
T1
0 μM
1 mM
Loop
G32
Bulge
U23

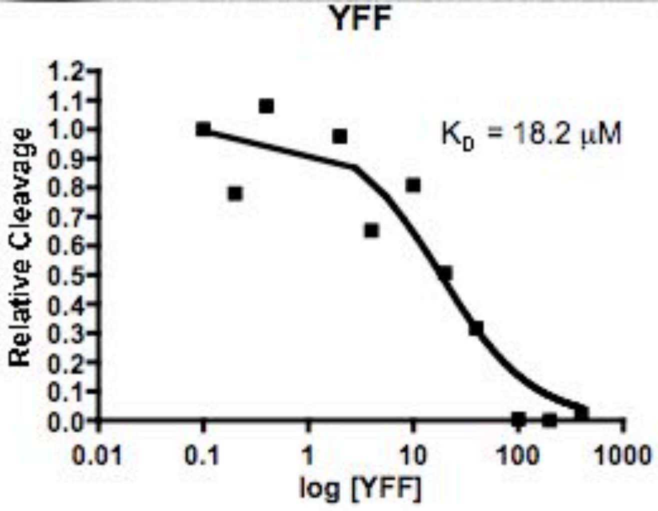
YFF
Relative Cleavage
log [YFF]
K_D = 18.2 μM
1.2
1.1
1.0
0.9
0.8
0.7
0.6
0.5
0.4
0.3
0.2
0.1
0.0
0.01
0.1
1
10
100
1000

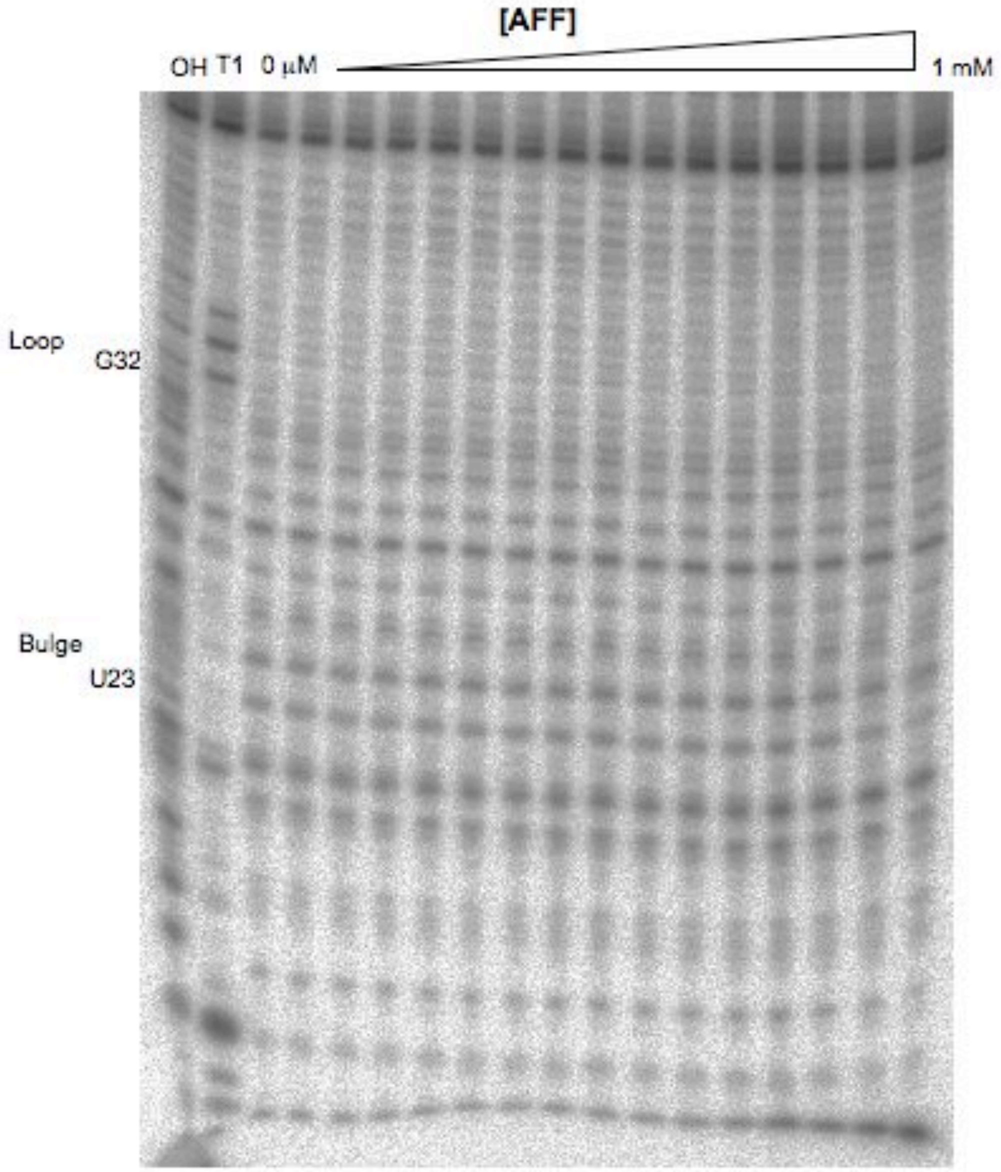
[AFF]
OH
T1
0 μM
1 mM
Loop
G32
Bulge
U23

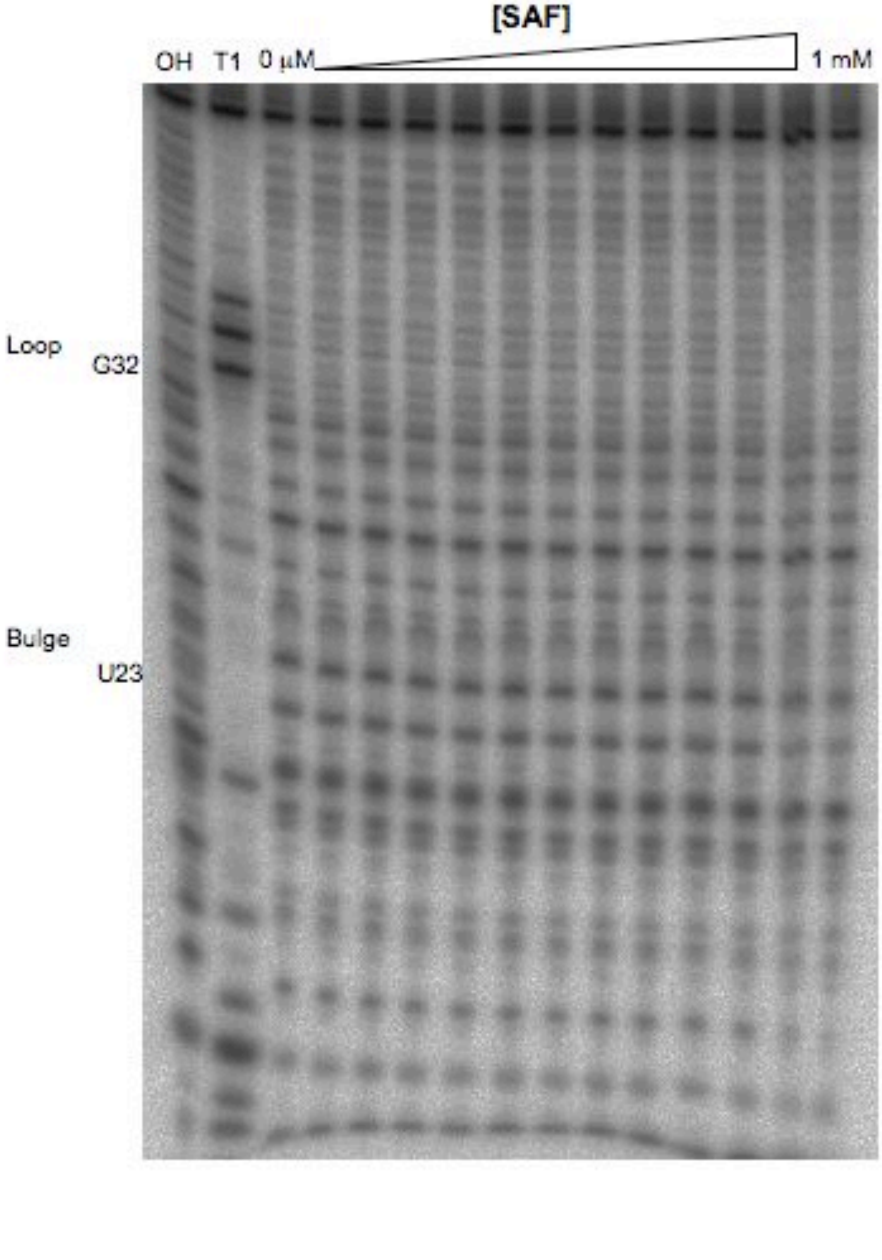
[SAF]
OH
T1
0 μM
1 mM
Loop
G32
Bulge
U23

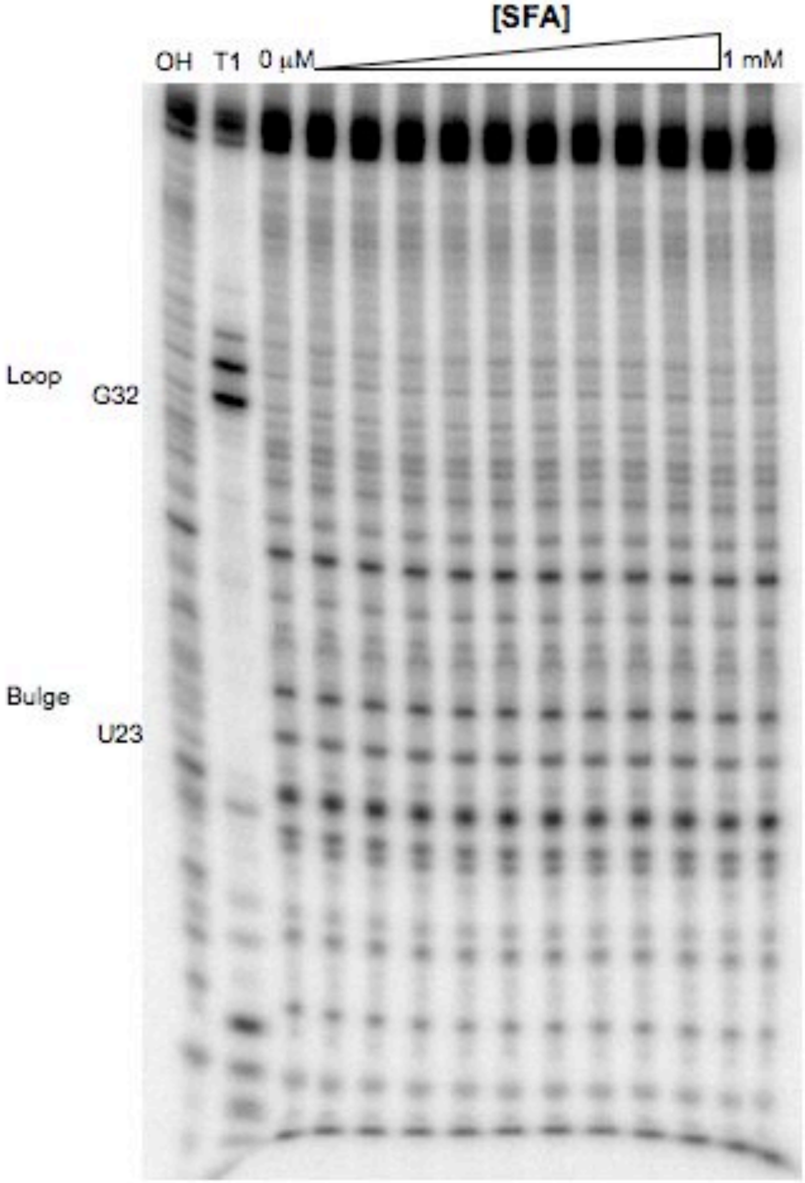
[SFA]
OH
T1
0 μM
1 mM
Loop
G32
Bulge
U23

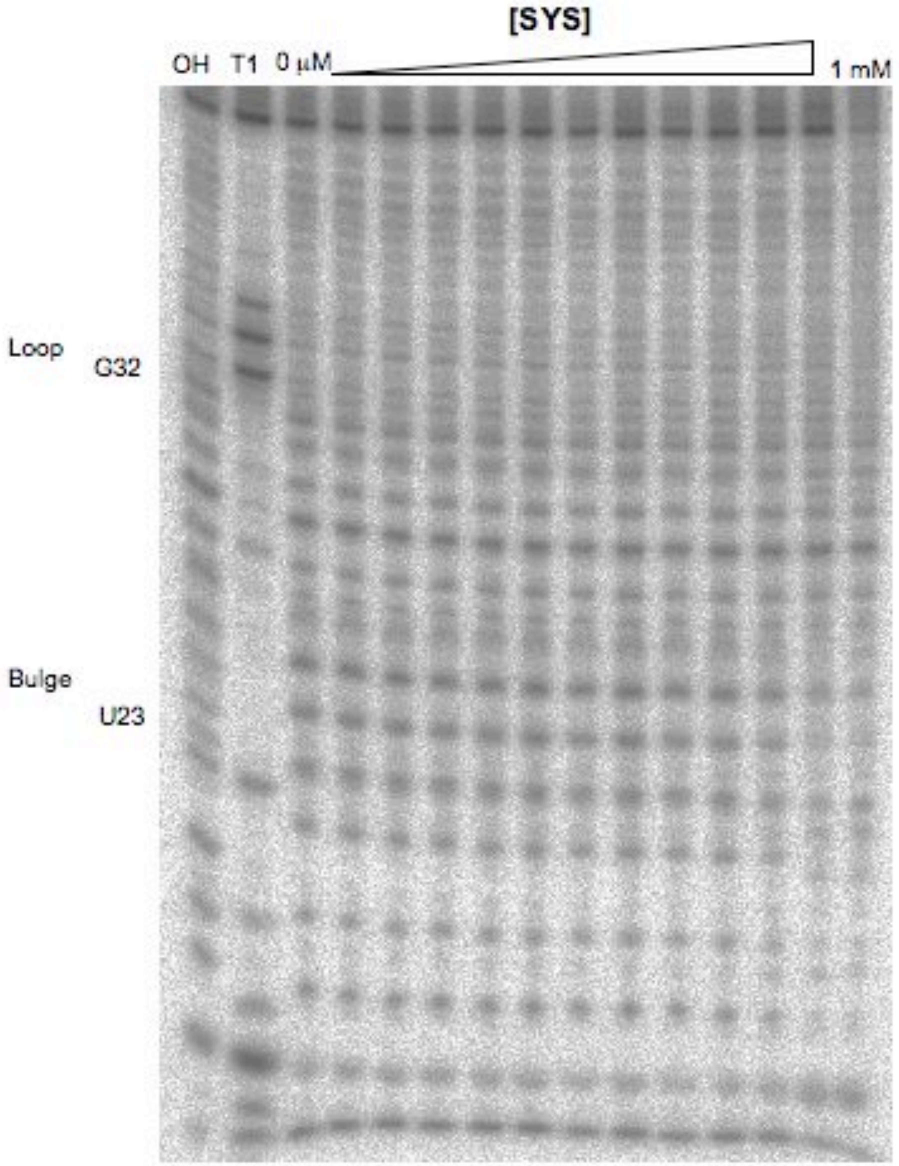
[SYS]
OH
T1
0 μM
1 mM
Loop
G32
Bulge
U23

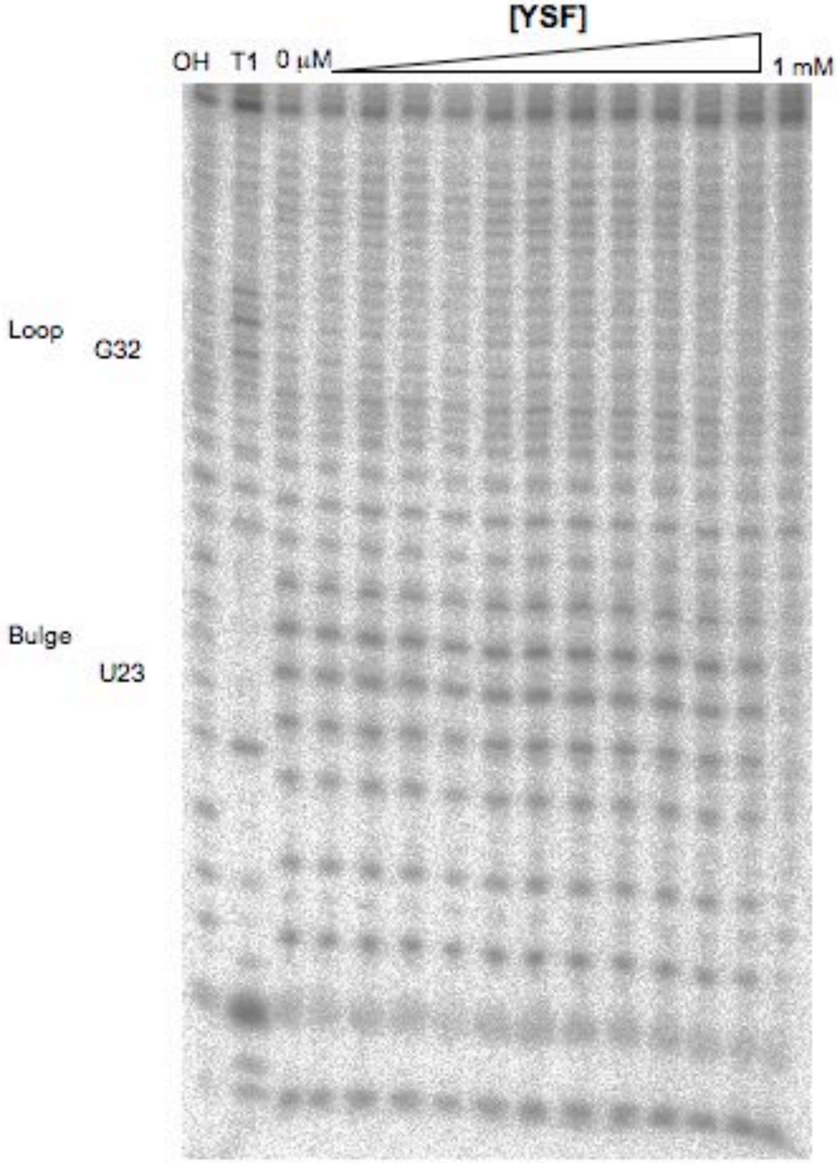
[YSF]
OH
T1
0 μM
1 mM
Loop
G32
Bulge
U23

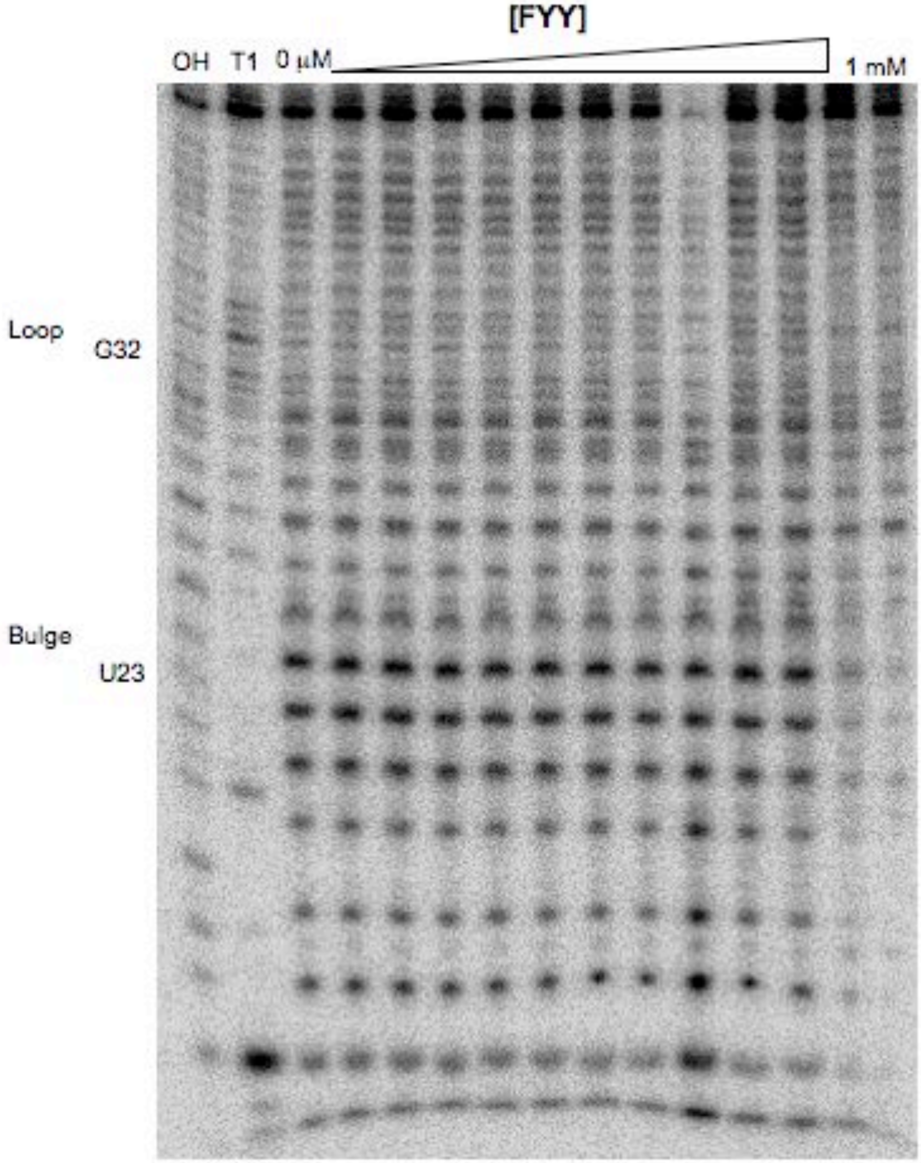
[FYY]
OH
T1
0 μM
1 mM
Loop
G32
Bulge
U23

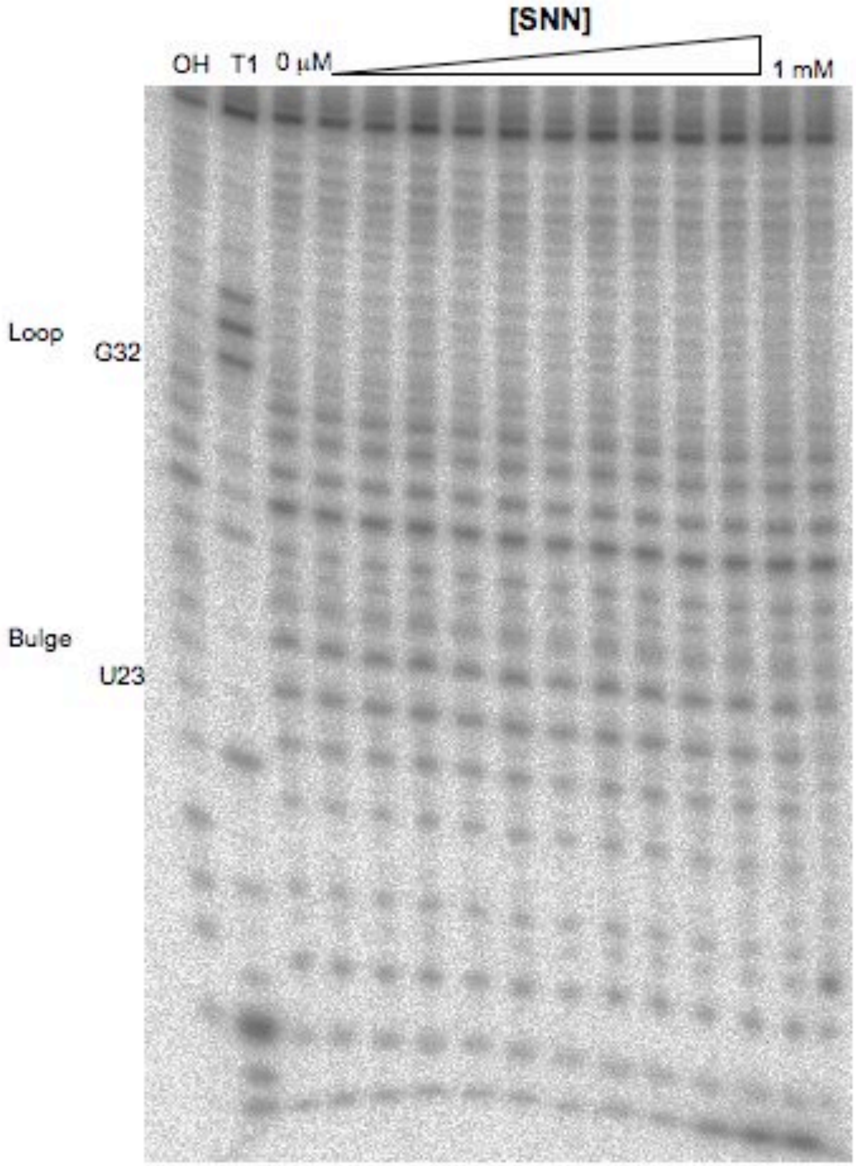
[SNN]
OH
T1
0 μM
1 mM
Loop
G32
Bulge
U23

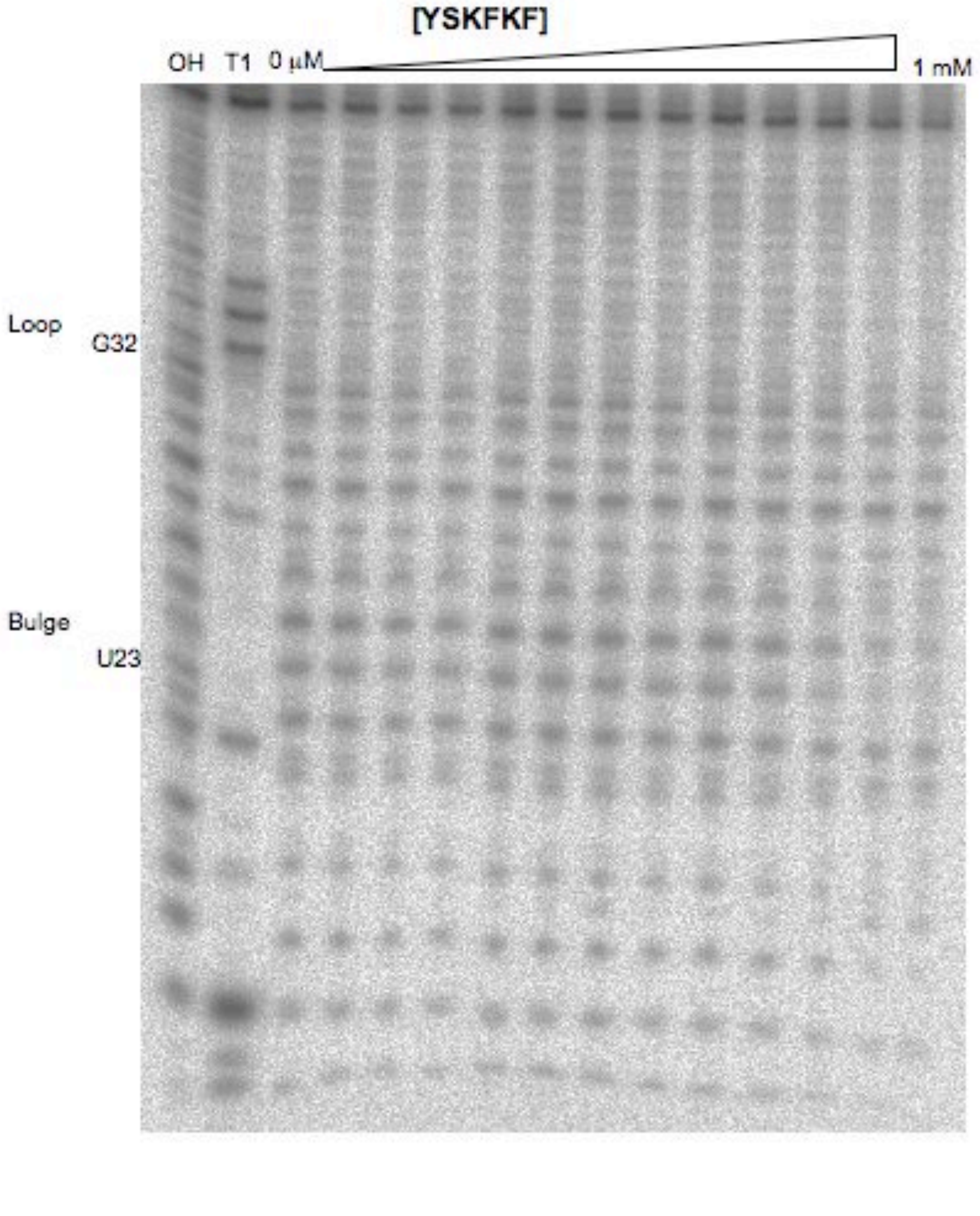

[YSKFKF]
OH
T1
0 μM
1 mM
Loop
G32
Bulge
U23

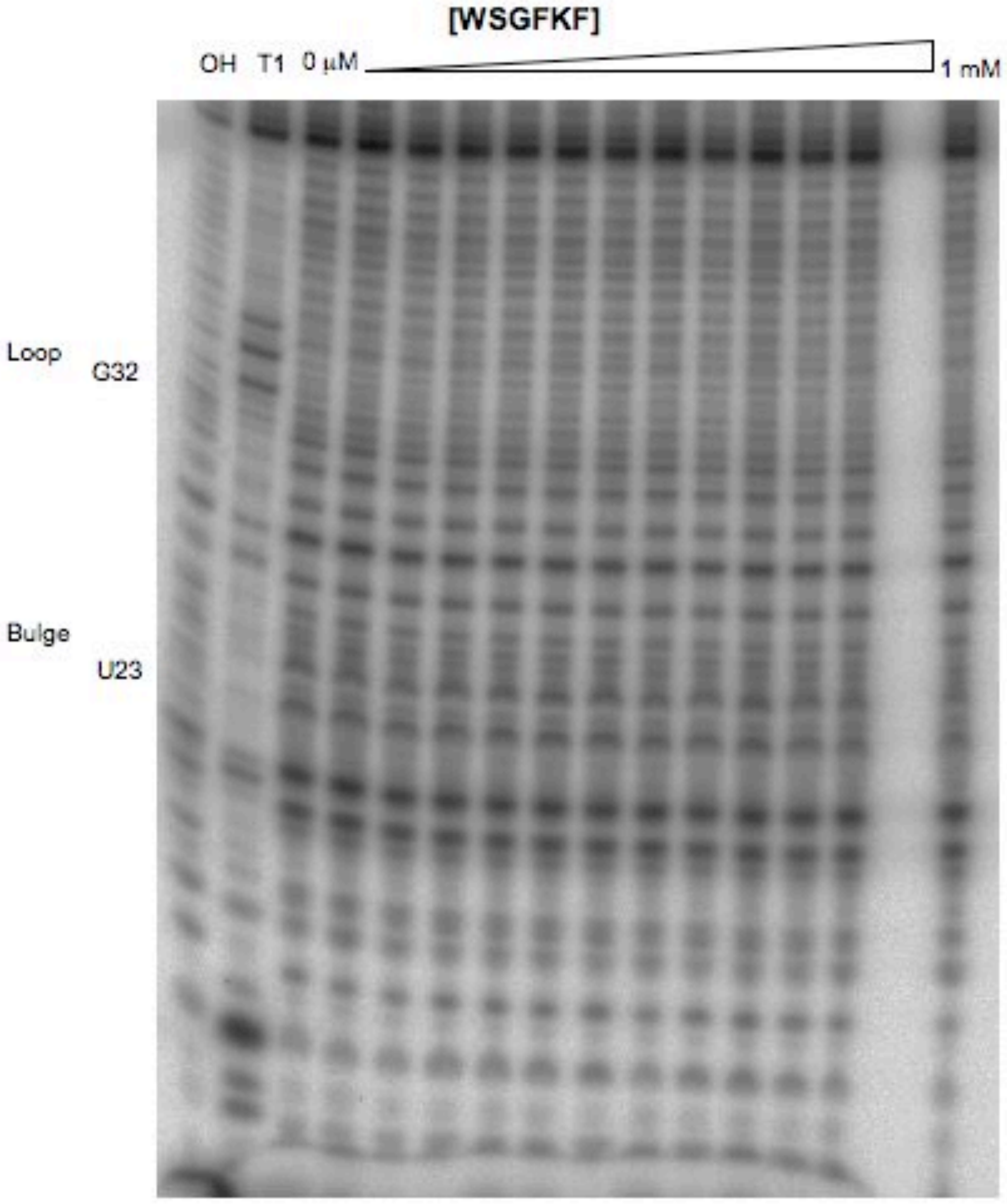
[WSGFKF]
OH
T1
0 μM
1 mM
Loop
G32
Bulge
U23

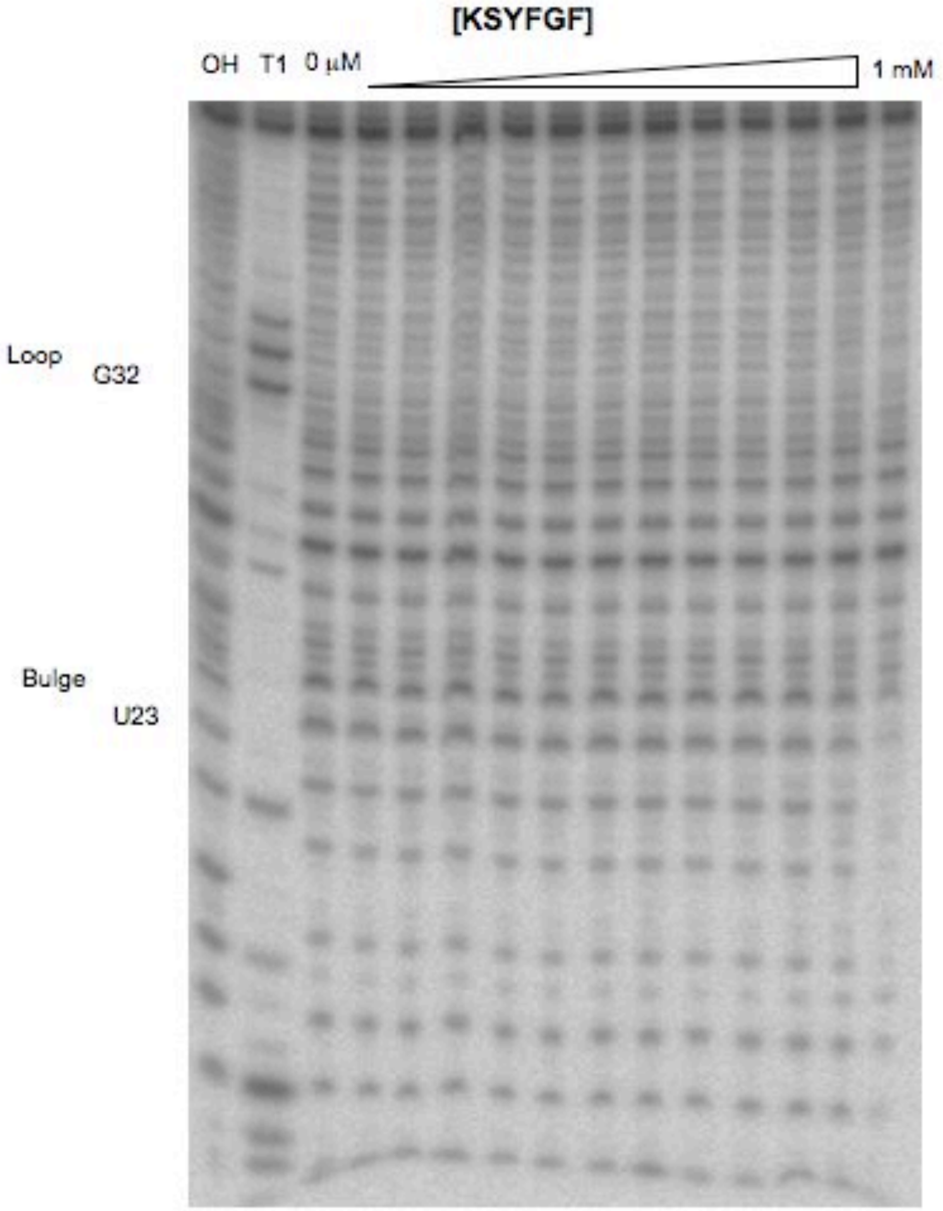

[KSYFGF]
OH
T1
0 μM
1 mM
Loop
G32
Bulge
U23

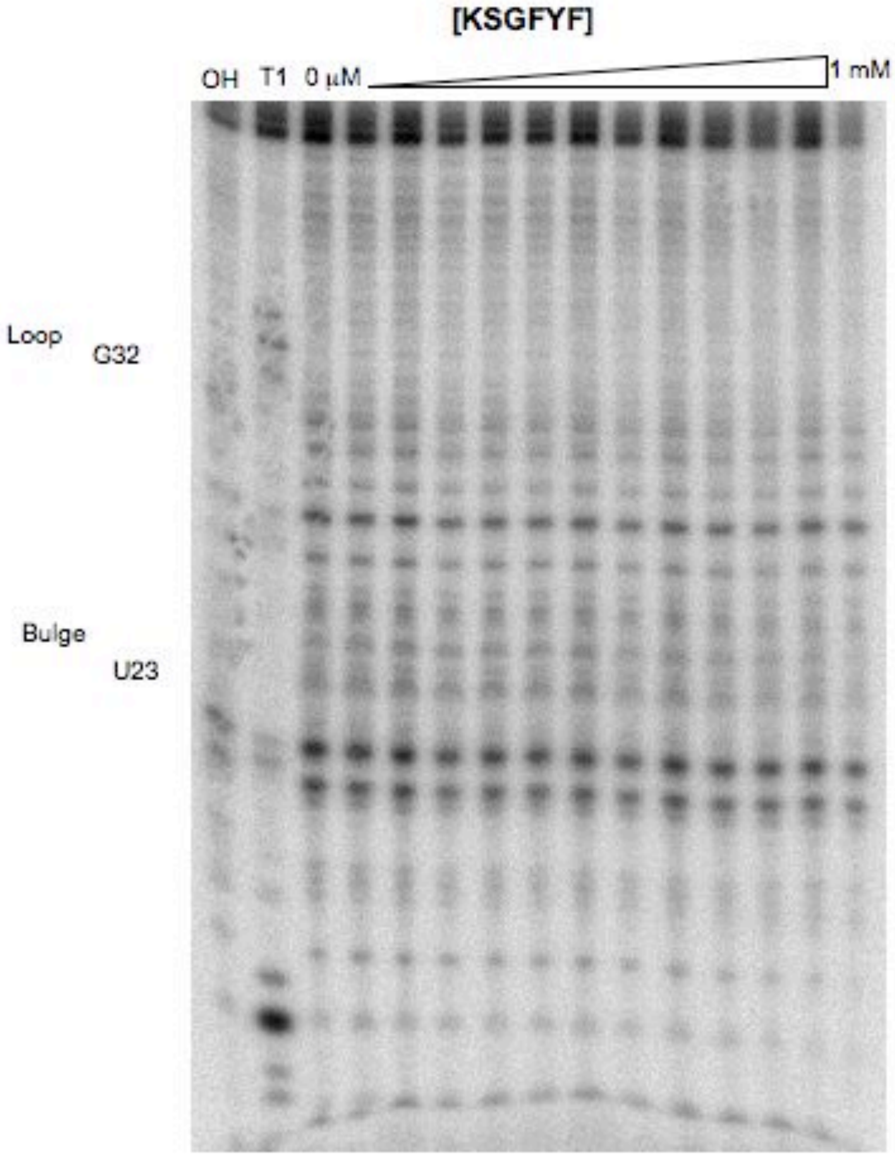
[KSGFYF]
OH
T1
0 μM
1 mM
Loop
G32
Bulge
U23

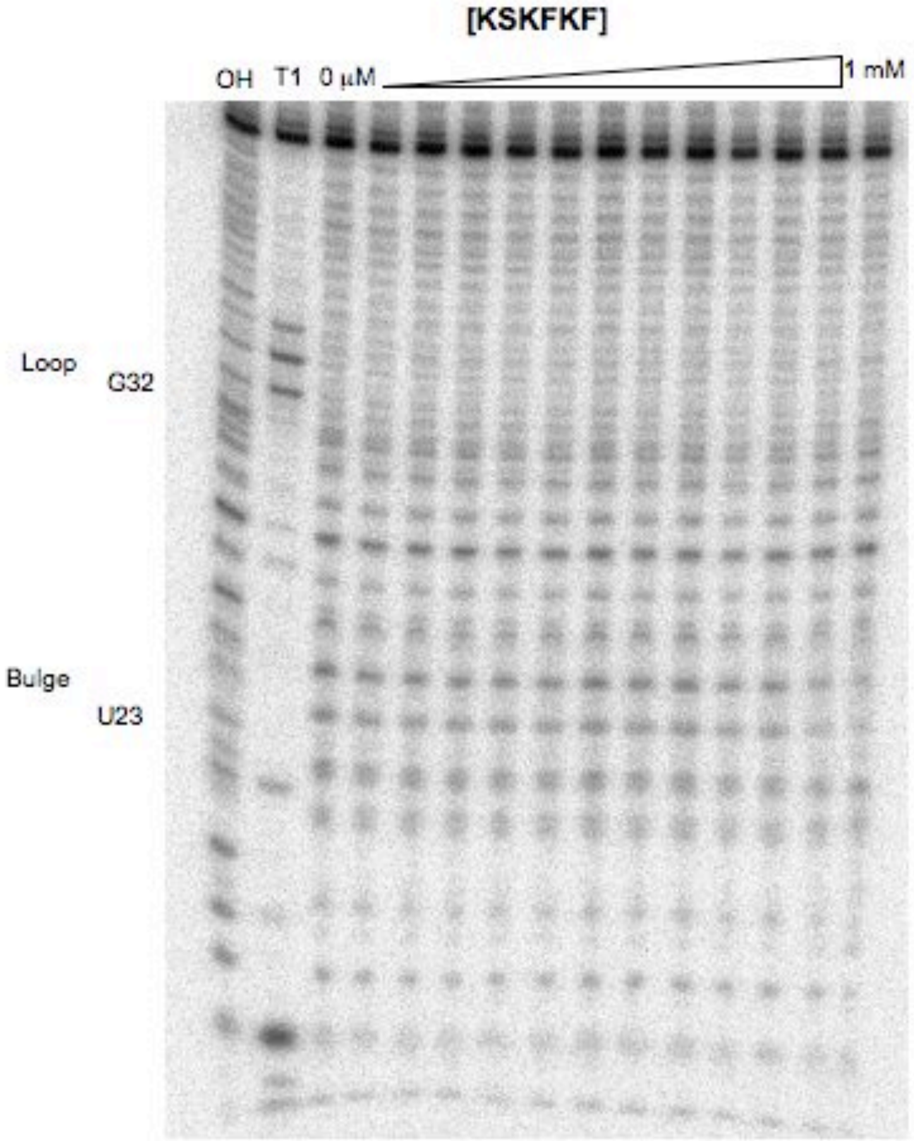
[KSKFKF]
OH
T1
0 μM
1 mM
Loop
G32
Bulge
U23
